# Data-Driven AI Approach for Optimizing Processes and Predicting Mechanical Properties of Boron Nitride Nanoplatelet-Reinforced PLA Nanocomposites

**DOI:** 10.3390/polym18020185

**Published:** 2026-01-09

**Authors:** Sundarasetty Harishbabu, Joy Djuansjah, P. S. Rama Sreekanth, A. Praveen Kumar, Borhen Louhichi, Santosh Kumar Sahu, It Ee Lee, Qamar Wali

**Affiliations:** 1 School of Mechanical Engineering, VIT-AP University, Besides A.P. Secretariat, Amaravati 522237, Andhra Pradesh, India; 2 College of Engineering, Imam Mohammad Ibn Saud Islamic University (IMSIU), Riyadh 11432, Saudi Arabia; 3 Department of Mechanical Engineering, Easwari Engineering College, Chennai 600089, Tamil Nadu, India; 4 Engineering Sciences Research Center (ESRC), Deanship of Scientific Research, Imam Mohammad Ibn Saud Islamic University (IMSIU), Riyadh 11432, Saudi Arabia; 5 Faculty of Artificial Intelligence and Engineering, Multimedia University, Cyberjaya 63100, Malaysia; 6 Centre for Smart Systems and Automation, COE for Robotics and Sensing Technologies, Multimedia University, Cyberjaya 63100, Malaysia

**Keywords:** injection molding, process parameters, ANOVA, machine learning

## Abstract

This research explores the optimization of mechanical properties and predictive modeling of polylactic acid (PLA) reinforced with boron nitride nanoplatelets (BNNPs) using data-driven machine learning (ML) models. PLA-BNNP composites were fabricated through injection molding, with a focus on how key processing parameters influence their mechanical performance. A Taguchi L27 orthogonal array was applied to assess the effects of BNNP composition (0.02 wt.% and 0.04 wt.%), injection temperature (135–155 °C), injection speed (50–70 mm/s), and pressure (30–50 bar) on properties such as tensile strength, Young’s modulus, and hardness. The results indicated that a 0.04 wt.% BNNP loading improved tensile strength, Young’s modulus, and hardness by 18.6%, 32.7%, and 20.5%, respectively, compared to pure PLA. Taguchi analysis highlighted that higher BNNP concentrations, along with optimal injection temperatures, improved all mechanical properties, although excessive temperatures compromised tensile strength and modulus, while enhancing hardness. Analysis of variance (ANOVA) revealed that injection temperature was the dominant factor for tensile strength (68.88%) and Young’s modulus (86.39%), while BNNP composition played a more significant role in influencing hardness (78.83%). Predictive models were built using machine learning (ML) models such as Random Forest Regression (RFR), Gradient Boosting Regression (GBR), and Extreme Gradient Boosting (XGBoost). Among the ML models, XGBoost demonstrated the highest predictive accuracy, achieving R^2^ values above 98% for tensile strength, 92–93% for Young’s modulus, and 96% for hardness, with low error metrics i.e., Root Mean Square Error (RMSE), Mean Absolute Error (MAE), Mean Absolute Percentage Error (MAPE). These findings underscore the potential of using BNNP reinforcement and machine learning-driven property prediction to enhance PLA nanocomposites’ mechanical performance, making them viable for applications in lightweight packaging, biomedical implants, consumer electronics, and automotive components, offering sustainable alternatives to petroleum-based plastics.

## 1. Introduction

Injection molding is one of the most widely used methods for mass production due to its high precision, efficiency, and reliability. In this process, the material is melted and injected under high pressure into a mold, where it solidifies into the desired shape. This technique is particularly suitable for manufacturing complex components within short cycle times [1]. Polylactic acid (PLA), a biodegradable bioplastic derived from renewable biomass resources such as cornstarch and sugarcane, has gained significant attention as a sustainable alternative to petroleum-based polymers. PLA is non-toxic to plants and animals, and it offers good processability [2,3]. However, owing to its poorer tensile properties and hardness, it restricts its use in engineering and medical applications. To address these shortcomings, researchers have developed PLA composites by incorporating natural fibers (e.g., bamboo, flax, hemp) and mineral fillers (e.g., talc, calcium carbonate) [4,5]. These reinforcements enhance mechanical strength and hardness. They provide not only reliable performance but also an environmentally friendly alternative to conventional petroleum-derived plastics [6,7].

Several studies have investigated strategies to enhance the performance of PLA-based composites by incorporating fillers. Vasu et al. [8] demonstrated that adding 0.15 wt.% hybrid nanofillers (graphene + SiO_2_) to epoxy–PLA composites improved tensile strength, flexural strength, and fracture toughness, mainly due to enhanced filler–matrix interfacial bonding and resistance to crack propagation. Similarly, Yusoff et al. [9] reported that incorporating 30 wt.% tapioca starch increased PLA tensile strength from 7.0 MPa to 9.7 MPa. Ferdinánd et al. [10] highlighted that blending PLA with polyethylene terephthalate (PET) enhanced stiffness, while polyvinyl alcohol (PVA) improved impact resistance. In another study, Cao et al. [11] showed that adding 1–4 wt.% tin–lead alloy powder enhanced tensile and impact strengths by 23.5% and 36.8%, respectively. The use of agricultural by-products as reinforcements has also been explored. Andrzejewski et al. [12] found that ground buckwheat husk (BH) slightly increased PLA crystallinity from 3.5% to 4.2%, though higher loadings decreased strength due to increased porosity. Kryszak et al. [13] developed PLLA/HAp composites and identified 20 wt.% HAp as optimal, achieving a tensile strength of 55 MPa, modulus of 2.5 GPa, impact strength of 18.1 kJ/m^2^, and crystallinity of 48%, along with good cell viability and osteogenic potential. However, higher HAp content increased S. aureus adhesion. Xueyang et al. [14] reported that jute/PLA composites reinforced with silane-treated nano-SiO_2_ exhibited superior properties, with tensile strength of 57 MPa, flexural strength of 95 MPa, and impact strength of 4.5 kJ/m^2^, attributed to improved nanoparticle dispersion and stronger filler–matrix bonding. Nanofillers have shown remarkable potential in enhancing PLA performance. Bedi et al. [15] demonstrated that 1 wt.% graphene oxide (GO) increased tensile strength by ~30% and thermal stability by 15–20 °C, while 1 wt.% CNTs improved Young’s modulus by up to 50%. Likewise, 3 wt.% cellulose nanocrystals (CNCs) enhanced crystallinity and strength through better filler dispersion. Maidana et al. [16] reported that 2 wt.% pseudoboehmite (PB) combined with 0.5 wt.% GO improved strength by 35%, stiffness by 40%, and thermal resistance by 15 °C. Liesenfeld et al. [17] confirmed that the addition of graphene to PLA significantly improved mechanical properties, including a 33.2% increase in tensile strength, 171% increase in tensile stiffness, 11.8% increase in flexural strength, and 34% increase in hardness. Jamadon et al. [18] showed that 1 wt.% magnesium hydroxide (Mg(OH)_2_) produced the best flexural and impact strengths of 85.2 MPa and 3.1 kJ/m^2^, respectively. Recycled materials and polymer blends have also been explored. Sam–Daliri et al. [19] demonstrated that incorporating 40 wt.% recycled GFRPP in injection-molded MEX-printed parts yielded the highest tensile strength of 29 MPa, with injection molding showing better results due to reduced porosity and improved fiber distribution. Pivsa–Art et al. [20] identified an optimal PLA/PP blend (80% PLA, 20% PP, and 3% PP-g-MAH) that achieved a tensile strength of 41.2 MPa. Batakliev et al. [21] showed that incorporating 1 wt.% graphite nanoplatelets (GNPs) or MWCNTs enhanced tensile strength to 58.6 MPa, while also improving crystallinity and thermal resistance. Padhy et al. [22] optimized the impact strength of 3D-printed PEEK by adjusting print orientation, print density, and chamber temperature. Using multi-objective optimization methods, the study found that 87.7% print density, 50 °C chamber temperature, and XZ orientation resulted in optimal impact strength (86.5 kJ/m^2^), reduced print time (89 min), and lowered material usage (3.26 g). A regression model with an adjusted R^2^ of 51.37% was developed to predict impact strength. Similarly, Moradi et al. [23] observed that PLA reinforced with 2 wt.% mesoporous nano-bioactive glass (m-nBG) exhibited tensile strength of 37.14 MPa, flexural strength of 72.2 MPa, and the highest cell viability, though higher loadings reduced strength. Nanoparticles have also proven effective in enhancing mechanical properties. For instance, Nikzad et al. [24] reported that 1.0–2.0 nm silica nanoparticles increased tensile strength by 25%, improved Young’s modulus by 18%, and raised the glass transition temperature by 10 °C (up to 135 °C). Similarly, Joy et al. [25] developed epoxy/MABS nanocomposites reinforced with hexagonal boron nitride (h-BN), where the formulation containing 5 wt.% MABS and 0.5 wt.% h-BN (M5BN0.5) achieved superior performance, showing exceptionally high impact resistance (~5534.7 J/m^2^), improved tensile strength, and enhanced viscoelastic properties, making it suitable for structural applications.

Although extensive research has explored various fillers—such as CNTs, GO, SiO_2_, CNCs, and natural fibers—for improving PLA composites, the incorporation of boron nitride nanoplatelets (BNNPs) remains relatively underexplored. BNNPs were selected for their high mechanical strength, low density, and excellent dispersion properties. These characteristics contribute to increased tensile strength and Young’s modulus in PLA. The wide surface area of BNNPs enhances the interaction with the PLA matrix, improving overall mechanical performance by increasing stiffness and reducing deformation under stress [26]. The novelty of this study lies in systematically evaluating boron nitride nanoplatelets (BNNPs), which possess exceptional mechanical strength, chemical stability, and biocompatibility, yet their impact on PLA’s mechanical performance has not been comprehensively explored. The objective of this work is to investigate PLA–BNNP composites through a hybrid strategy that integrates experimental characterization with AI-driven, data-driven approaches. The Taguchi method is applied for experimental design and optimization, while ANOVA is used to identify the most significant processing parameters. In parallel, AI-driven machine learning models are employed to predict mechanical properties with high accuracy. This integrated methodology establishes a robust pathway for developing high-performance and sustainable PLA–BNNP composites.

## 2. Materials and Methods

### 2.1. Materials

**Figure 1:**
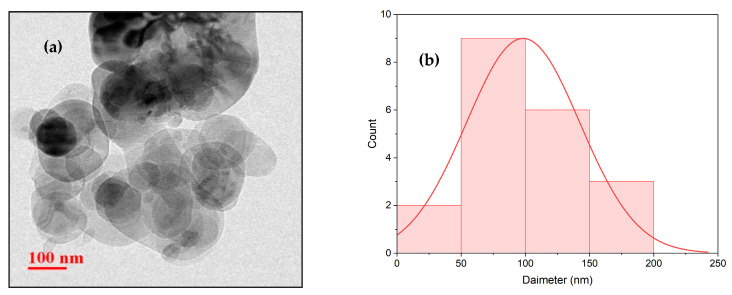
(**a**) TEM image of BNNP; (**b**) particle diameter distribution histogram of BNNP.

The polylactic acid (PLA) granules used in this study possessed a density of 1.20–1.30 g/cm^3^ and a melt flow rate (MFR) of 1 g/10 min and were supplied by Banka BioLoo Limited, Hyderabad, India. Platelet-shaped boron nitride nanoplatelets (BNNPs) with a molecular weight of 24.82 and particle size below 100 nm were procured from Nano Research Elements, Kurukshetra, India. As shown in Figure 1a, the BNNPs exhibited a flat, layered, platelet-like morphology under transmission electron microscopy (TEM) (Talos F200X, Thermo Fisher Scientific Inc., Waltham, MA, USA) and functioned at 200 kV. The particle size distribution, presented in Figure 1b, was obtained by measuring particle diameters from transmission electron microscopy (TEM) images using the ImageJ software v1.54 (National Institute of Health, Bethesda, MD, USA). The resulting histogram indicates a near-normal distribution centered around 100 nm, with the majority of particles falling in the 100 nm range and a small fraction of larger aggregates appearing above this size.

### 2.2. Fabrication of Samples

**Figure 2:**
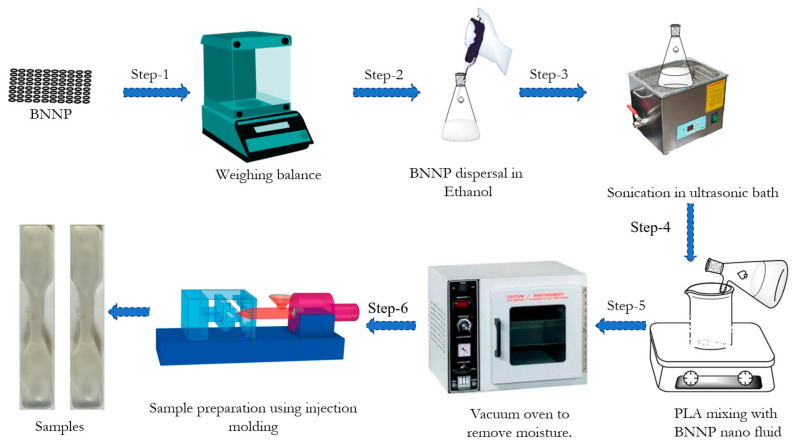
Steps involved in fabricating the composite sample.

Composite samples were fabricated using polylactic acid (PLA) as the base polymer, reinforced with boron nitride nanoplatelets (BNNPs) at loadings of 0.02 and 0.04 wt.%. The fabrication process is outlined in Figure 2. Initially, PLA and BNNP were precisely weighed using a digital balance (Shimadzu ATX-224, Shimadzu Co., Kyoto, Japan). The BNNPs were dispersed in ethanol and subjected to ultrasonication to break agglomerates and achieve uniform distribution. Subsequently, PLA granules were gradually introduced into the BNNP–ethanol dispersion while stirring on a magnetic hot plate, facilitating interaction between the polymer and the nanoplatelets. The mixture was then dried in a vacuum oven (GR-58, Nano Tec, Chennai, India) at 70 °C for 24 h to remove residual ethanol and moisture. The dried PLA/BNNP composite was processed in a semiautomated horizontal injection-molding machine (Deesha Impex Pvt. Ltd., Ahmedabad, India), with a barrel temperature range of 145–180 °C, packing pressure of 50 MPa, and a packing time of 10 s. Injection temperature, speed, and pressure play a critical role in determining the tensile strength, Young’s modulus, and hardness of PLA-BNNP composites. Proper temperature ensures better nanoplatelet dispersion, while speed influences material flow, and pressure helps achieve uniformity and better bonding [27,28]. Standard tensile specimens were prepared in accordance with ASTM guidelines.

### 2.3. Design of Experiments (DOEs) with Taguchi

The Taguchi method is a robust statistical approach widely applied in fabrication and engineering to design experiments efficiently and optimize process parameters. By employing orthogonal arrays, it enables the systematic evaluation of multiple factors and their levels while minimizing the number of experimental trials, thereby ensuring high-quality and reliable outcomes. In this study, optimization was carried out for composition and injection-molding parameters, including temperature, injection speed, and injection pressure, each considered at three levels, as summarized in Table 1. A full factorial design with four factors at three levels would require 81 experimental runs (3^4^ = 81). However, the Taguchi method, using an L27 orthogonal array, reduced the number of required trials to 27, without compromising the reliability of the analysis [28,29]. To evaluate the experimental results and determine the most significant parameters, both the mean values and the signal-to-noise (S/N) ratios were analyzed. The S/N ratio, a key metric in the Taguchi method, reflects the robustness of the process and helps in minimizing variability in the measured responses. As the objective of this study was to enhance the mechanical properties, the “larger-the-better” criterion for the S/N ratio was adopted. This criterion is mathematically expressed by Equation (1) [30].

(1) $$\frac{S}{N}\;ratio=-10\log_{10}\left[\frac{1}{n}\sum_{i=1}^{n}\frac{1}{{O_{i}}^{2}}\right]$$

### 2.4. Mechanical Testing

#### 2.4.1. Tensile Testing

Tensile tests for neat PLA and PLA/BNNP composites were conducted in accordance with ASTM D638 [31] using a Universal Testing Machine (UTM, H10KL, Tinius Olsen India Pvt. Ltd., Noida, India), as shown in Supplementary Figure S1. The specimens were subjected to uniaxial tensile loading at a constant crosshead speed of 2 mm/min, and the stress–strain data obtained were used to calculate tensile strength and Young’s modulus. To improve reliability and minimize variability, each test was performed five times, and the average values were reported [31,32]. After mechanical testing, fractographic analysis of the fractured specimens was performed using a Scanning Electron Microscope (ZEISS EVO 10, Oberkochen, Germany)) to examine surface morphology and failure mechanisms. Before imaging, the fractured surfaces were sputter-coated with a thin gold layer to ensure conductivity, and observations were carried out at the required accelerating voltage.

#### 2.4.2. Hardness Testing

Hardness testing of neat PLA and PLA/BNNP composites was carried out in accordance with ASTM E384 [33] using a Micro Vickers Hardness Tester (MC-AT, Fine Spavy Associates & Engineers Pvt. Ltd., Miraj, India), as illustrated in Supplementary Figure S2. Each specimen was subjected to microindentation with a diamond pyramidal indenter having a face angle of 136° and under a load of 0.5 kg for a dwell time of 10 s [34,35]. After unloading, the diagonals of the indentations were measured using an optical microscope, and the Vickers Hardness Number (VHN) was calculated using Equation (2) [36]. All tests were performed at room temperature, and the average of five indentations was reported to ensure accuracy and repeatability.

(2) $$\mathrm{HV}=2\;\frac{F\;\sin\frac{136}{2}}{l^{2}}$$

Here, HV is Vickers hardness, F is the applied load in kgf, and l is the average length of the two diagonals.

### 2.5. Statistical Analysis by Analysis of Variables (ANOVA)

Analysis of Variance (ANOVA) is a widely used statistical technique for evaluating the influence of different factors on experimental outcomes. In this study, ANOVA was applied to the results obtained from the Taguchi design of experiments to assess the statistical significance of the parameters listed in Table 1 on the mechanical properties of neat PLA and PLA/BNNP composites, including tensile strength, Young’s modulus, and hardness. The analysis was conducted using Minitab-2021 at a 95% confidence level. The F-ratio and corresponding *p*-values were examined to determine the relative influence of each factor, with parameters considered statistically significant when the *p*-value was less than 0.05 [37,38].

### 2.6. Machine Learning (ML)

**Figure 3:**
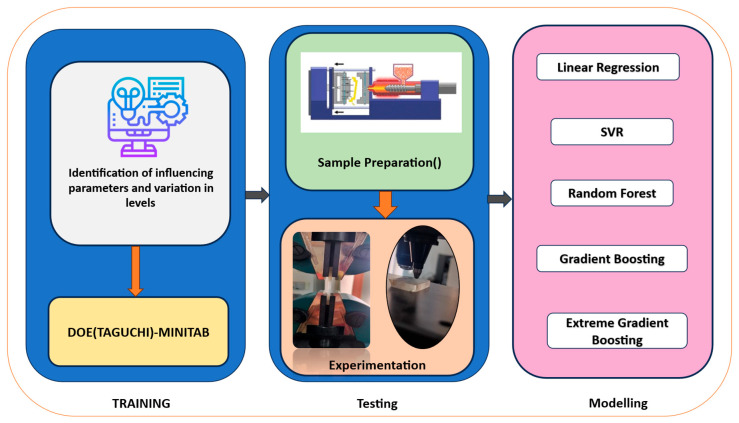
Machine learning process chart.

Machine learning (ML) is a modern and efficient approach for optimizing the mechanical properties of materials, offering clear advantages over conventional trial-and-error methods. By learning the underlying relationships between processing parameters and mechanical responses, ML techniques enable accurate prediction and optimization of outcomes while significantly reducing experimental effort. As illustrated in Figure 3, machine learning (ML) is a modern and efficient approach for optimizing the mechanical properties of materials, offering clear advantages over conventional trial-and-error methods. By learning the underlying relationships between processing parameters and mechanical responses from experimental data, ML techniques enable accurate prediction and optimization of outcomes while significantly reducing experimental effort. As illustrated in Figure 3, ML models serve as powerful tools for guiding material design and process optimization. In the present study, ML approaches were employed to optimize the injection-molding process parameters. The models were trained using an initial dataset generated from systematically designed injection molding, in which the key processing parameters and the corresponding mechanical properties were used as input–output pairs. After training and validation, the developed ML models were applied to analyze the experimental results and identify optimal process conditions using the following techniques [39].

#### 2.6.1. Linear Regression

Linear Regression (LR) serves as a baseline algorithm to establish the linear relationship between the independent variables (processing parameters listed in Table 1) and the dependent variables, including tensile strength, Young’s modulus, and hardness of neat PLA and PLA/BNNP composites. The model assumes a linear correlation among the variables and seeks to minimize the residual sum of squares between the observed and predicted responses. In this study, LR was implemented with all input features standardized to enhance numerical stability. The dataset, obtained from the Taguchi experimental design, was divided into training and testing sets to evaluate and validate model performance, as described by Equations (3) and (4) [40].

General Linear Regression Model

(3) $$\bar{O}=a_{0}+a_{1}p_{1}+a_{2}p_{2}+a_{3}p_{3}+a_{4}p_{4}$$

Here, $p_{1},p_{2},p_{3},p_{4}$ are the composition, temperature, injection speed, and injection pressure, respectively, and $a_{0},a_{1}$, $a_{2},a_{3}$ are the model coefficients.

Model Coefficient Estimation

(4) $$\bar{a}={\left(P^{T}P\right)}^{-1}P^{T}y$$

Here, P is the scaled input parameters matrix, and y is the scaled output parameters, such as tensile strength, Young’s modulus, and hardness.

#### 2.6.2. Support Vector Regression

Support Vector Regression (SVR) is a machine learning technique employed to predict and optimize the mechanical properties of PLA/BNNP composites under varying injection-molding conditions. SVR is particularly effective in capturing nonlinear relationships between multiple input parameters and output responses, even with limited data availability. In this study, SVR constructs a regression function to model the relationship between the processing parameters listed in Table 1 and mechanical responses, including tensile strength, Young’s modulus, and hardness of neat PLA and PLA/BNNP composites. The core components of SVR—regression function, kernel mapping, and optimization—enable accurate modeling of complex, nonlinear behaviors. The mathematical formulation of these components is detailed in Equations (5)–(7) [41].

The basic form of the SVR model is

(5) $$F\left(P\right)=w^{T}p+b$$

Here, $P{\left[Composition,Temperature,Injection\;Speed,Injection\;Pressure\right]}^{T}$ is the input vector, w is the weight vector, b is the bias term, and $w^{T}p$ is the dot product between w and P.

The epsilon-insensitive loss function is

(6) $$L\left(O,f\left(P\right)\right)=\left\{\begin{array}{c} 0\;if\;\left|O-f\left(P\right)\right|<€\\ \left|y-f\left(p\right)\right|-€\;\mathrm{other}\;\mathrm{wise}\; \end{array}\right.$$

Here, y is the actual output variable (tensile strength, Young’s modulus, and hardness), $f\left(P\right)$ is the predicted output function, and € is the insensitive margin (tolerance zone).

Objective optimization is

(7) $$\underset{w,b,d,d^{*}}{\min}\left(\frac{1}{2}{\left\|w\right\|}^{2}+C\sum_{i=1}^{n}d_{i}+{d^{*}}_{i}\right)$$

which is subject to

$$O_{i}-w^{T}P_{i}-b\leq €+d_{i}$$

$$w^{T}P_{i}+b-O_{i}\leq €+{d^{*}}_{i}$$

$$d_{i},{d_{i}}^{*}\geq 0$$

Here $d_{I},{d_{i}}^{*}$—slack variables for positive and negative deviations beyond €—is a regularization parameter controlling the penalty of errors, and n is the total number of data points.

#### 2.6.3. Random Forest Regression

Random Forest Regression (RFR) is a widely adopted machine learning algorithm for regression tasks due to its high accuracy, robustness, and capability to model complex relationships between input and output variables. It is particularly effective in handling nonlinear data and minimizing overfitting. RFR is an ensemble learning technique that constructs multiple decision trees, where each tree is trained on a bootstrapped sample of the training dataset and considers a random subset of input features at each split. This approach reduces variance and enhances predictive accuracy, robustness, and generalization of the model. The mathematical formulation of RFR is described in Equations (8) and (9) [42].

Define a dataset as follows:

(8) $${D=\{(P_{i},O_{i})\}}_{i=1}^{\;n}$$

Here, $P_{I}$ is [composition, temperature, injection speed, injection pressure], and $O_{I}$ is [tensile strength, Young’s modulus, hardness].

For the given input factors P, the Random Forest Regression consists of N regression trees.

$f_{1}\left(P\right)$, $f_{2}\left(P\right),f_{3}\left(P\right)$, …….$f_{n}\left(P\right)$ predicts the output $\bar{O}$ as the average of the individual tree predictions.

(9) $$\bar{O}\left(P\right)=\frac{1}{N}\sum_{n=1}^{N}f_{n}\left(P\right)$$

Here, $\bar{O}\left(P\right)$ is the predicted value for the input P, N is the total number of decision trees in the forest, and $f_{n}\left(P\right)$ is the prediction from the $N^{th}$ tree.

#### 2.6.4. Gradient Boosting

Gradient Boosting Regression (GBR) is an ensemble machine learning technique designed for regression problems with complex and nonlinear relationships. It sequentially builds predictive models to minimize the loss function using gradient descent, correcting errors made by previous models. GBR combines multiple weak learners, typically shallow decision trees, into a strong predictive model, improving accuracy and reducing bias. Unlike Random Forest Regression, which relies on bagging and parallel training, GBR employs a boosting strategy, focusing on the most challenging cases in the dataset. This makes it particularly effective for modeling the mechanical behavior of composite materials based on multiple processing parameters. The underlying methodology is mathematically represented by Equations (10)–(13) [43], describing the additive model and gradient-based update mechanism.

Define a dataset as follows:

(10) $${D=\{(P_{i},O_{i})\}}_{i=1}^{\;m}$$

Here, $P_{I}$ is [composition, temperature, injection speed, injection Poessure], and $O_{I}$ is [tensile strength, Young’s modulus, hardness].

(11) $$F_{M}\left(P\right)=\sum_{m=1}^{m}R_{m}h_{m}\left(P\right)$$

The gradient descent update is as follows:

(12) $$F_{m}\left(P\right)=F_{m-1}\left(P\right)+R_{m}h_{m}\left(P\right)$$

Here, $R_{m}$ is the learning rate or weight applied to each tree, M is the total number of iterations (trees), and $h_{m}\left(P\right)$ is the $m^{th}$ weak learner (decision tree).

h_m_ is trained to fit the pseudo-residuals

(13) $$h_{m}\approx r_{i}^{m}=\left[\frac{\partial l\left(O_{i},F_{m-1}\left(P_{i}\right)\right)}{\partial F_{m-1}\left(P_{i}\right)}\right]$$

Here, $r_{i}^{m}$ is the pseudo-residual for data point i at boosting iteration m, $O_{i}$ is the actual target value, $F_{m-1}\left(P_{i}\right)$ is prediction from the model after m−1 iterations, and $\partial l\left(O_{i},F_{m-1}\left(P_{i}\right)\right)$ is the loss function (e.g., mean squared error).

#### 2.6.5. Extreme Gradient Boosting (XG-Boost)

Extreme Gradient Boosting (XGBoost) is a widely adopted machine learning algorithm in both research and industry due to its versatility, stability, and strong capability in preventing overfitting. Unlike standard gradient boosting methods, XGBoost employs a more regularized model formalization by incorporating L1 and L2 penalties into the loss function, enhancing generalization and model robustness. In this study, XGBoost is applied to predict the mechanical properties of PLA/BNNP composites, including tensile strength, Young’s modulus, and hardness, using composition, injection temperature, injection speed, and injection pressure as input features. The model builds additive functions by minimizing a regularized objective function through regression trees, mathematically represented in Equations (14) and (15) [44,45].

(14) $$L\left(t\right)=\sum_{i=1}^{n}\left(l(O_{i}{{\bar{O}}_{i}}^{t}\right)+\sum_{m=1}^{t}\varphi \left(f_{m}\right)$$

(15) $$\varphi \left(f\right)=\beta T+\frac{1}{2}\delta {\left\|\omega \right\|}^{2}$$

Here, l is a differentiable loss function, $f_{m}$ represents an individual regression tree at iteration m, T is the number of leaves in the tree, ω is the vector of scores on leaves, and β,δ are regularization parameters that penalize model complexity.

## 3. Results and Discussion

### 3.1. Mechanical Testing

#### 3.1.1. Tensile Testing Results

**Figure 4:**
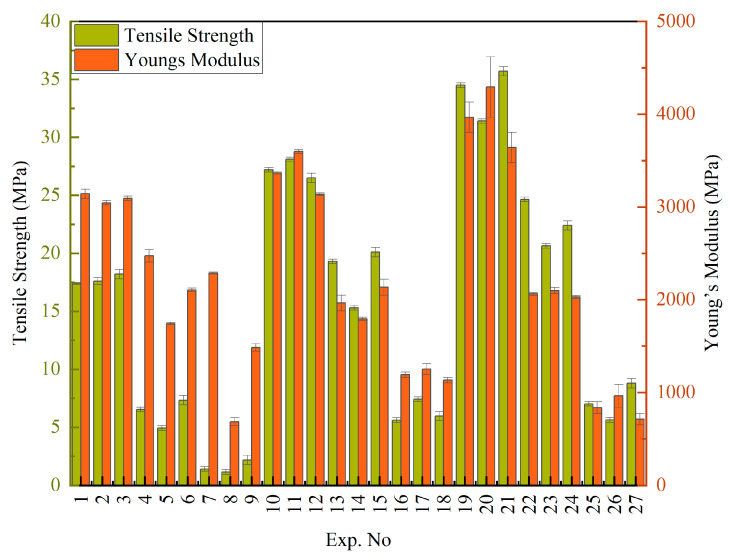
Experimentally obtained tensile strength and Young’s modulus.

Figure 4 presents the tensile strength and Young’s modulus values obtained using the L27 orthogonal array, which was designed based on the methodology of Design of Experiments (DOEs). The Young’s modulus varied between 712 MPa and 3966 MPa, while the tensile strength ranged from 1.27 MPa to 35.7 MPa. These variations highlight the sensitivity of the mechanical properties to changes in process parameters such as temperature, composition, injection speed, and injection pressure [46,47]. To further analyze the experimental results presented in Table 2, the Minitab 2021 software was employed to determine the optimal levels for each process parameter.

**Figure 5:**
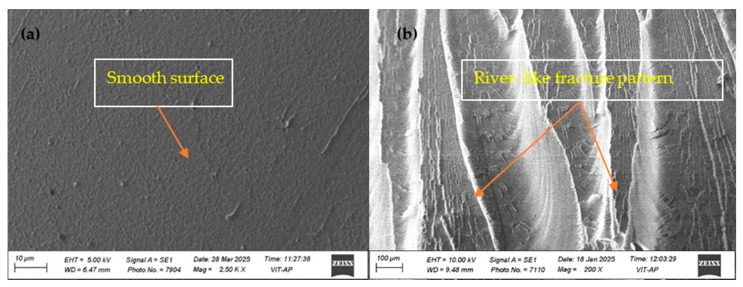
Fractography of (**a**) pure PLA; (**b**) 0.04 wt.% PLA/BNNP composite, processed under optimized injection-molding parameters.

Figure 5a,b shows SEM micrographs of the fractured surfaces for pure PLA and the 0.04 wt.% PLA/BNNP composite, all fabricated under optimized injection-molding conditions. The pure PLA sample, as shown in Figure 5a, exhibits typical brittle fracture features characterized by a smooth and featureless surface, indicating rapid crack propagation with minimal plastic deformation. Such morphology is commonly observed in brittle polymers, where failure occurs with limited energy absorption [48]. The 0.04 wt.% BNNP composite in Figure 5b exhibits a more pronounced river-like fracture pattern and rougher fracture topology, signifying enhanced interfacial bonding and efficient load transfer between the filler and the matrix. This improved morphology contributes to greater energy absorption and increased toughness of the composite material [49].

#### 3.1.2. Hardness Testing

**Figure 6:**
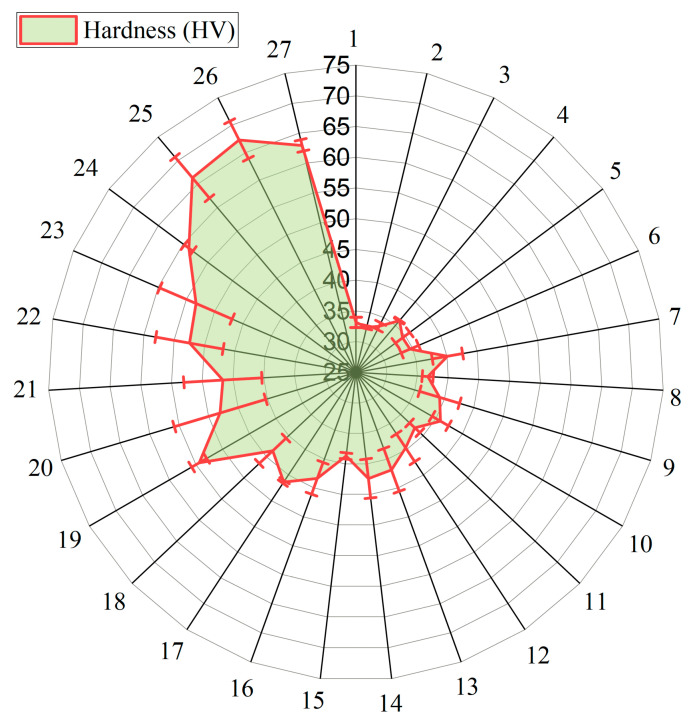
Experimentally obtained hardness.

The hardness of the PLA/BNNP composite samples was evaluated using the L27 orthogonal array to investigate the effects of process parameters. As shown in Figure 6, the hardness measured in Exp-1 was 33.03 HV, while the maximum value of 67.3 HV was observed in Exp-26, indicating a substantial improvement, as summarized in Table 3. This variation reflects the combined influence of critical process parameters, including BNNP content, processing temperature, injection pressure, and injection speed. The results show that the incorporation of BNNP significantly enhances the hardness of the composites. Furthermore, optimizing the processing conditions contributes to improved microstructure and densification, which in turn positively affects hardness.

### 3.2. Statistical Analysis

#### 3.2.1. Taguchi Analysis

To perform the Taguchi analysis, the experimentally obtained values of tensile strength, Young’s modulus, and hardness and they were used to calculate the signal-to-noise (S/N) ratios, as presented in Table 2 and Table 3. The optimization of process parameters based on the Taguchi method for each of these outcomes is discussed in the following sections.

##### Tensile Testing

The response tables are presented in Table 4, indicating the influence of each factor on tensile strength. Results indicate that temperature is the most influential [50,51], as observed by the highest delta values of 16.22 and 21.27. Composition comes next, with delta values of 10.19 and 12.66, indicating a moderate influence. Injection speed shows a lower but still considerable effect, ranking third place with delta values of 3.05 and 4.34. Injection pressure, in contrast, shows the least impact with the lowest delta values of 2.62 and 2.76, ranking fourth. Based on these findings, one may conclude that the changes in temperature and composition significantly influence tensile strength, while the changes in injection pressure are relatively negligible. This pattern is reflected both in the S/N ratio and in the mean response, as per the “larger-the-better” criterion.

**Figure 7:**
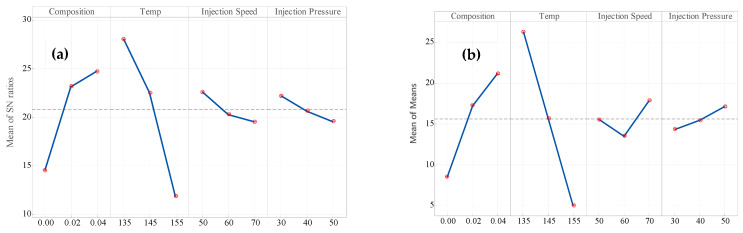
Main effect plot of (**a**) mean of S/N ratio for Young’s modulus, (**b**) mean of mean for Young’s modulus (larger is better).

The main effect plots in Figure 7a,b for the mean of S/N ratio and the mean of the mean response indicate that temperature is the most critical factor. In both plots, as the temperature increases from 135 °C to 155 °C, a sharp decrease in tensile strength indicates a strong negative correlation. Composition is the second-most crucial factor. An increasing trend is observed where higher levels of composition are succeeded by improved tensile strength, although there are slight fluctuations between levels. Injection speed impacts moderately. Through the S/N ratio plot, tensile strength reduces gradually when speed is higher, while a slight increase is seen at greater speeds (70 mm/s) in the mean plot, which reflects a nonlinear tendency. The impact of injection pressure is the least. Both plots show minimal variation with pressure levels, implying a secondary role in tensile strength variation. Temperature and composition are typically the controlling tensile strength parameters, with injection speed and pressure having relatively minor influences.

##### Young’s Modulus

The most significant influencing factor on Young’s modulus is depicted in Table 5. The test results show temperature to have the most significant influence [52], and the maximum delta values of 10.71 and 2304. The second-most important factor is the injection pressure, with delta values of 1.92 and 457, implying a moderate impact. Injection speed ranked third with 1.02 and 369 for the delta values. By contrast, composition is the least influential, with the lowest delta values of 0.49 and 115, ranking fourth. The results indicate that varying temperature and injection speed will have a significant impact on the Young’s modulus, regardless of composition, which has a comparatively minor role [53]. This trend holds for both the S/N ratio and the mean response, according to the “larger-the-better” criterion.

**Figure 8:**
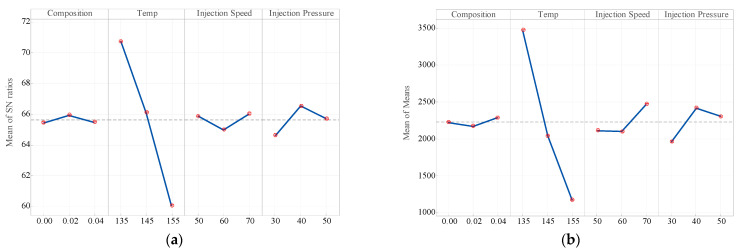
Main effect plot of (**a**) mean of S/N ratio for Young’s modulus, (**b**) mean for Young’s modulus (larger is better).

Figure 8a,b, which depicts the main effect plots of the mean of signal-to-noise (S/N) ratio and mean of mean response for Young’s modulus, shows that temperature is confirmed as the most significant factor influencing Young’s modulus. As the temperature increases from 135 °C to 155 °C, Young’s modulus decreases sharply, indicating a strong negative correlation. Injection pressure is identified as the second-most impactful parameter, with variations in pressure levels noticeably affecting Young’s modulus. Injection speed exhibits a moderate influence, showing a general decline in Young’s modulus with increasing speed, but the increase observed the highest speed (70 mm/s), suggesting a nonlinear behavior. Composition has the least effect on Young’s modulus, with only minor variations observed across different levels.

##### Hardness

The process parameters influencing hardness are presented in Table 6, based on the “larger-the-better” criterion for both the S/N ratio and the mean response. The results show that the composition is the most influential parameter, exhibiting the highest delta values of 3.99 and 21.13. Temperature ranks second, with delta values of 1.60 and 8.78, with a moderate impact. Injection speed shows a noticeable but lesser influence, ranking third with delta values of 0.43 and 3.58. Among all the process parameters, injection pressure has the least effect, with the lowest delta values of 0.41 and 3.48, placing it as fourth. Based on this analysis, it is observed that composition and temperature variations significantly affect hardness [54], while the remaining process parameters play a relatively minor role.

**Figure 9:**
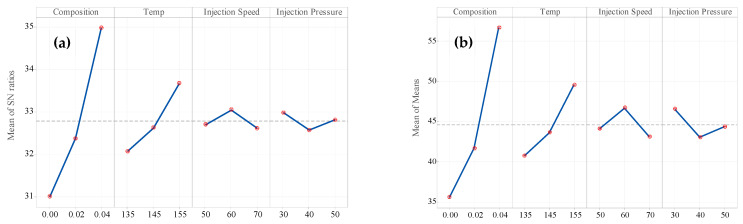
Main effect plot of (**a**) mean of S/N ratio for Young’s modulus; (**b**) mean of mean for hardness (larger is better).

Figure 9a,b shows the mean compelling plots of both signal-to-noise (S/N) ratio and mean response for the Young’s modulus. It indicates that composition has the most effect on hardness. As the composition rises, hardness increases significantly, demonstrating a strong positive correlation. The second-most significant factor of hardness is temperature, where increasing temperature tends to enhance hardness, albeit less so than composition. Injection speed has a moderate effect; only slight variations in hardness are observed across speeds, with a slight peak at 60 mm/s, indicative of nonlinear behavior. The injection pressure has the minimum influence, with minimal fluctuation in hardness across the tested pressure range. Composition and temperature are the prevailing parameters overall, with injection speed and injection pressure contributing relatively less.

#### 3.2.2. Analysis of Variance (ANOVA)

##### Tensile Testing

**Figure 10:**
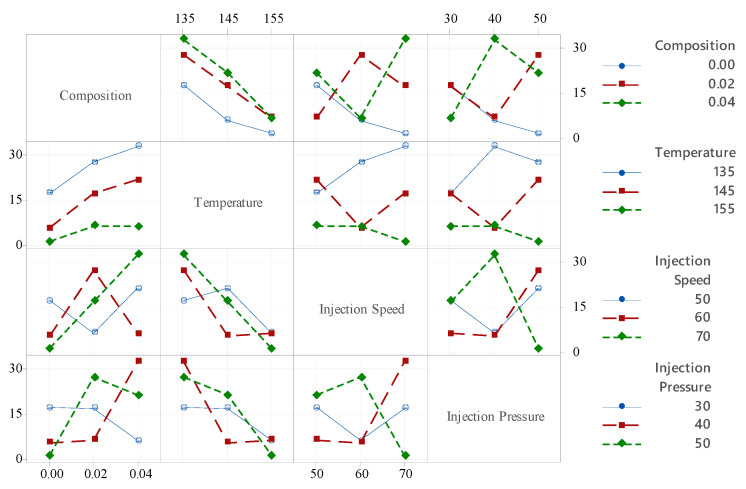
Interaction plot of process parameters for tensile strength.

The ANOVA analysis was conducted to evaluate the impact of injection-molding parameters, including composition (P1), temperature (P2), injection speed (P3), and injection pressure (P4), on tensile strength, as summarized in Table 7. The results revealed that temperature had the largest effect, contributing 68.88%, followed by composition (25.61%), injection speed (2.87%), and injection pressure (1.46%). The *p*-values for all factors were significantly less than 0.05, confirming their statistical significance: temperature (*p*-value = 0.007 × 10^−13^), composition (*p*-value = 0.004 × 10^−9^), injection speed (*p*-value = 0.006 × 10^−2^), and injection pressure (*p*-value = 0.005). The corresponding F-values were also high, with temperature having the highest F-value of 423.89, followed by composition with 157.60, injection speed with 17.65, and injection pressure with 7.26, further emphasizing the dominant role of temperature in influencing tensile strength. The model’s goodness of fit is reflected in the R-squared value of 98.54% and the adjusted R-squared value of 97.89%, demonstrating a strong correlation between the experimental and predicted tensile strengths and confirming the model’s predictive accuracy. These high R^2^ and adjusted R^2^ values demonstrate a strong correlation between the experimental and predicted results obtained from the regression equation (Equation (16)), indicating that the developed model provides an excellent fit for accurately predicting tensile strength. Figure 10 provides an interaction plot, showing how various process parameters influence the tensile strength at different levels of a categorical factor, such as composition, temperature, injection speed, and injection pressure, represented along the *x*-axis. The lines on the plot depict the levels of other factors, with the mean tensile strength displayed on the *y*-axis. The interaction plot for the tensile strength of PLA/BNNP composites shows that tensile strength generally decreases with increasing filler composition, dropping from ~30 MPa at 0.00 composition to ~15 MPa at 0.04 composition. Temperature has a nonlinear effect, with tensile strength peaking around 145 °C and declining at higher temperatures (155 °C), indicating potential thermal degradation. Injection speed exhibits a mixed influence, but moderate speeds (60 mm/s) tend to maintain or slightly improve tensile strength compared to lower (50 mm/s) and higher (70 mm/s) speeds. Injection pressure also affects tensile strength, where intermediate pressures (40 bar) often yield better results than very low (30 bar) or high (50 bar) pressures. Overall, the interaction plot highlights that optimizing these processing parameters is critical for maximizing the mechanical performance of the composites.

Regression Equation

Tensile strength = 15.663 − 7.140 P_1(0.00)_ + 1.612 P_1(0.02)_ + 5.528 P_1(0.04)_ + 10.626 P_2(135)_             + 0.023 P_2(145)_ − 10.649 P_2(155)_ − 0.122 P_3(50)_ − 2.107 P_3(60)_ + 2.229 P_3(70)_ − 1.294 P_4(30)_ − 0.179 P_4(40)_ + 1.473P_4(50)._   (16)

**Figure 11:**
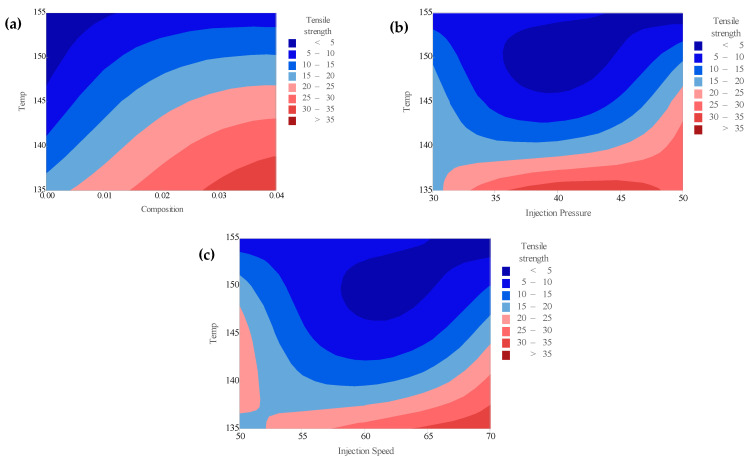
Counter plots of tensile strength vs. (**a**) temperature and composition, (**b**) temperature and injection speed, and (**c**) temperature and injection pressure.

The contour plots in Figure 11a–c depict the combined influence of temperature with composition, injection pressure, and injection speed, respectively, on the tensile strength of PLA/BNNP composites. In Figure 11a, at constant injection speed (60 mm/s) and pressure (40 bar), tensile strength increases steadily with both composition and moderate temperatures, exceeding 35 MPa when filler content is 0.03–0.04 wt.% and temperature is 135–145 °C. At low composition (pure PLA) and high temperature (155 °C), the strength remains below 5 MPa, highlighting the importance of filler-induced reinforcement and controlled thermal processing [55,56]. Figure 11b, generated at constant composition (0.04 wt.%) and injection speed (60 mm s^−1^), reveals that maximum tensile strength > 35 MPa occurs at 135–145 °C and higher injection pressures at 45–50 bar, whereas temperatures above 150 °C cause significant reductions, regardless of pressure, due to polymer chain degradation and reduced molecular alignment [57,58]. Figure 11c, at fixed composition (0.04 wt.%) and pressure (40 bar), shows an optimal range of 135–145 °C and 65–70 mm/s for peak tensile strength, with performance dropping at higher temperatures [59,60]. The pronounced vertical gradients and curvature of the contour lines in Figure 11a–c confirm that temperature is the dominant variable, while processing parameters modulate its effect.

##### Young’s Modulus

The ANOVA analysis was conducted to evaluate the impact of injection-molding parameters, including composition, as shown in Table 8. The results revealed that temperature (P2) had the largest effect, contributing 86.39%, followed by injection pressure (P4) with 3.60%, injection speed (P3) with 2.79%, and composition (P1) with 0.21%. The *p*-values for temperature (0.007 × 10^−8^), injection speed (0.048), and injection pressure (0.024) were all significantly less than 0.05, confirming their statistical significance. However, the *p*-value for composition 0.766 was greater than 0.05, indicating that it had a negligible effect on Young’s modulus. The corresponding F-values were also high, with temperature having the highest F-value of 110.95, followed by injection pressure with 4.62, injection speed with 3.59, and composition with 0.27, further emphasizing the dominant role of temperature in influencing Young’s modulus. The model’s goodness of fit is reflected in the R-squared value of 92.99% and the adjusted R-squared value of 89.88%, demonstrating a strong correlation between experimental and predicted results, confirming the model’s predictive accuracy. These high R^2^ and adjusted R^2^ values demonstrate a strong correlation between the experimental and predicted results obtained by the regression equation, as shown in Equation (17), indicating that the developed model is an excellent fit for accurately predicting the Young’s modulus.

Regression Equation:

Young’s modulus = 2230.4 − 2.100P_1(0.00)_ − 56.300P_1(0.02)_ + 58.400P_1(0.04)_ + 1244.900P_2(135)_
          − 185.900P_2(145)_ − 1059.000P_2(155)_ − 114.200P3_(50)_ − 127.200P_3(60)_         + 241.400P_3(70)_ − 265.700 P_4(30)_ + 191.100 P_4(40)_ + 74.600 P_4(50)_ (17)

**Figure 12:**
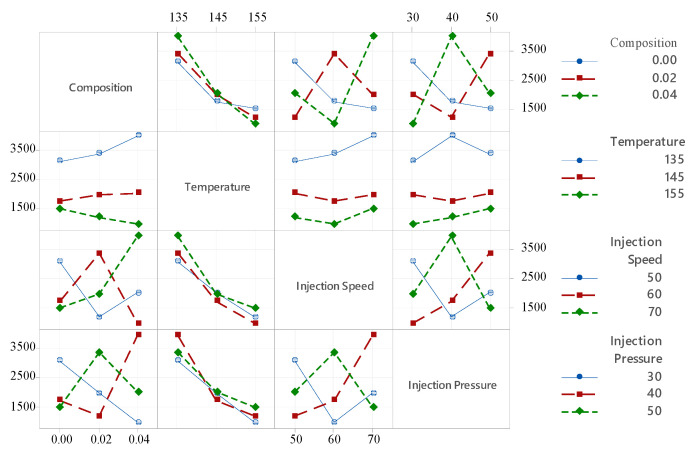
Interaction plot of Young’s modulus.

**Figure 13:**
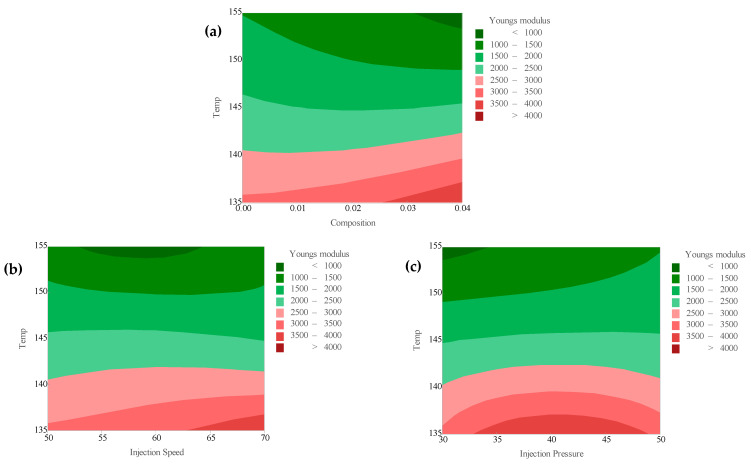
Counter plots of Young’s modulus vs. (**a**) temperature and composition, (**b**) temperature and injection speed, and (**c**) temperature and injection pressure.

Figure 12 provides an interaction plot, illustrating how different process parameters influence the Young’s modulus at various levels of categorical factors such as composition, temperature, injection speed, and injection pressure, which are displayed along the x-axis. The plotted lines represent the mean tensile strength values across different settings of these factors, shown on the y-axis. In the case of PLA/BNNP composites, the tensile strength exhibits a general downward trend with increasing filler composition, decreasing from approximately 3500 MPa at 0.00 composition to around 1500 MPa at 0.04 composition. Temperature shows a nonlinear behavior, where tensile strength initially increases and peaks at about 145 °C before declining at higher temperatures (155 °C), possibly due to thermal degradation effects. Injection speed has a varied impact: a moderate injection speed of 60 mm/s tends to maintain or slightly improve tensile strength compared to lower (50 mm/s) or higher (70 mm/s) speeds, where performance drops. Injection pressure also influences tensile strength, with intermediate pressures around 40 bar generally achieving better mechanical properties compared to very low (30 bar) or high (50 bar) pressures. Overall, the interaction plot emphasizes the importance of finely tuning the processing parameters to optimize the tensile strength of PLA/BNNP composites. The contour plots in Figure 13a–c reveal the effects of process parameters on Young’s modulus for PLA-based composites. In Figure 13a, higher temperatures above 145 °C reduce the modulus, while increasing filler composition from 0.00 to 0.04 wt.% slightly enhances stiffness, especially at lower temperatures (135–145 °C). Figure 13b shows that higher injection speeds (65–70 mm/s) combined with low temperatures (135–145 °C) yield modulus values above 3500 MPa, whereas elevated temperatures lower the modulus regardless of speed. Similarly, Figure 13c indicates that low temperatures (135–145 °C) with higher injection pressures (45–50 MPa) result in maximum modulus values above 4000 MPa, while temperatures above 145 °C consistently lead to values below 2000 MPa. The steep vertical gradients and curvature of the contour lines in all three plots confirm temperature as the primary controlling factor, with other processing parameters modulating its influence.

##### Hardness

The ANOVA analysis was conducted to evaluate the impact of injection-molding parameters— temperature, injection speed, and injection pressure, including composition—on hardness, as summarized in Table 9. The results revealed that composition had the largest effect, contributing 78.83%, followed by temperature with 13.36%, injection speed with 2.27%, and injection pressure with 2.05%. The *p*-values for composition (0.004 × 10^−10^), temperature (0.006 × 10^−4^), injection speed (0.011), and injection pressure (0.015) were all significantly less than 0.05, confirming their statistical significance. The corresponding F-values were also high, with composition having the highest F-value of 203.66, followed by temperature with 34.51, injection speed with 5.88, and injection pressure with 5.30, further emphasizing the dominant role of composition in influencing hardness. The model’s goodness of fit is reflected in the R-squared value of 96.52% and adjusted R-squared value of 94.97%, demonstrating a strong correlation between experimental and predicted results, confirming the model’s predictive accuracy. These high R^2^ and adjusted R^2^ values demonstrate a strong correlation between the experimental and predicted results obtained by the regression equation, as shown in Equation (18), indicating that the developed model is an excellent fit for accurately predicting hardness [61].

Regression Equation:

Hardness = 44.644 − 9.078P_1(0.00)_ − 2.978P_1(0.02)_ + 12.056P_1(0.04)_ − 3.878P_2(135)_ − 1.022P_2(145)_        + 4.900P_2(155)_ − 0.533P_3(50)_ + 2.056P_3(60)_ − 1.522P_3(70)_ + 1.878P_4(30)_ − 1.600P_4(40)_
    − 0.278 P_4(50)_                            (18)

**Figure 14:**
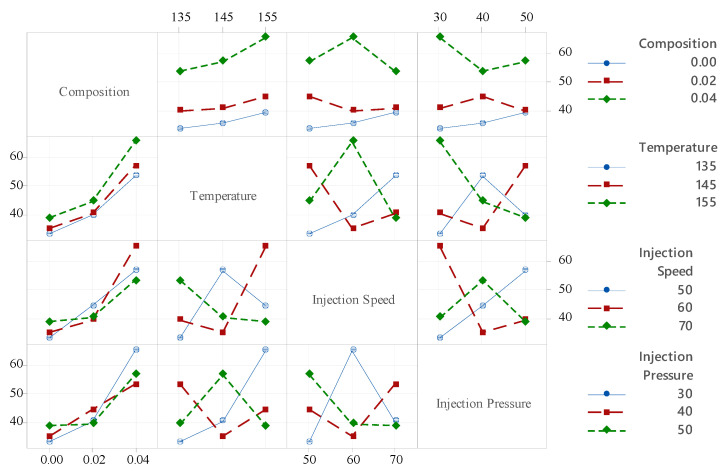
Interaction plot of hardness.

Figure 14 presents an interaction plot demonstrating how different process parameters influence the hardness at various levels of categorical factors such as composition, temperature, injection speed, and injection pressure, shown along the x-axis. The plotted lines represent the mean hardness values across different factor settings, with the hardness displayed on the y-axis. For PLA/BNNP composites, hardness tends to increase with higher filler composition, rising from around 40% at 0.00 composition to over 60% at 0.04 composition, suggesting improved ductility with filler addition. Temperature exhibits a positive trend, with hardness increasing as the temperature rises from 135 °C to 155 °C, likely due to enhanced polymer chain mobility at higher temperatures. The effect of injection speed is more complex: while moderate speeds (60 mm/s) sometimes show hardness, variability is observed across different compositions and pressures. Injection pressure influences hardness notably, where an intermediate pressure (40 bar) generally achieves higher hardness compared to both lower (30 bar) and higher (50 bar) pressures. Overall, the interaction plot highlights that optimizing these processing parameters is crucial for enhancing the ductility and mechanical flexibility of PLA/BNNP composites.

**Figure 15:**
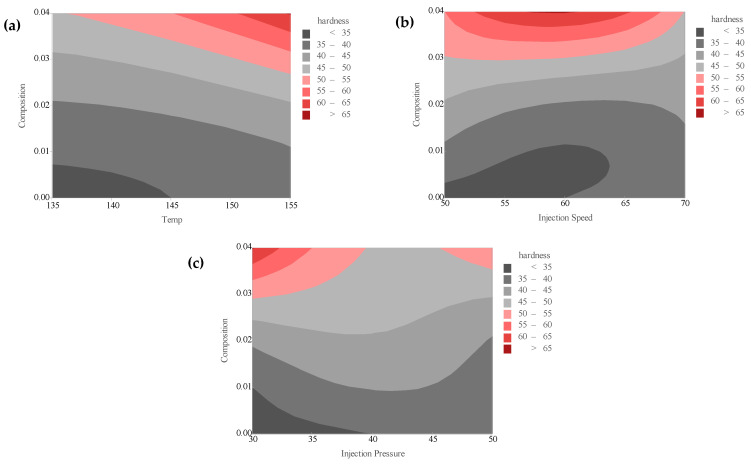
Counter plots of hardness vs. (**a**) composition and temperature, (**b**) composition and injection speed, and (**c**) composition and injection pressure.

Figure 15a–c illustrates the combined effects of filler composition with temperature, injection speed, and injection pressure, respectively, on the hardness of PLA/BNNP composites. In Figure 15a, with injection speed fixed at 60 mm/s and pressure at 40 bar, hardness for pure PLA (0.00 composition) remains around 36–38 across the temperature range, while at 0.04 composition it rises sharply to 54.4–67.3, indicating that temperature variation has only a minor effect. Figure 15b, at a constant temperature of 145 °C and pressure of 40 bar, shows hardness near 35 for pure PLA at all injection speeds, increasing to 54.4–67.3 at 0.04 composition, confirming the negligible influence of injection speed. Figure 15c, with temperature at 145 °C and speed at 60 mm/s, reveals hardness close to 35 for pure PLA and increasing to 54.4–67.3 at 0.04 composition, with injection pressure having minimal impact. These results confirm that filler composition is the primary factor governing hardness in PLA/BNNP nanocomposites.

Supplementary Figure S3a,b illustrates the statistical analysis of injection-molding parameters based on *p*-values and F-values, respectively. Supplementary Figure S3a shows that temperature has the lowest *p*-value (0.004 × 10^−9^), indicating it has the most statistically significant impact on tensile strength, followed by composition (*p*-value 0.004 × 10^−9^) for hardness. Injection speed and injection pressure exhibit moderately higher *p*-values (0.006 × 10^−2^ and 0.005), suggesting they are less significant compared to temperature. Supplementary Figure S3b reveals that temperature has the highest F-value (423.89) for tensile strength, confirming its dominant effect on this property. Injection pressure shows a strong influence on hardness (F-value 7.26), while injection speed has a moderate effect on tensile strength (F-value 17.65). Composition mainly influences hardness, as reflected in the F-value of 157.60. These findings emphasize the importance of temperature in optimizing tensile strength and injection pressure for hardness and provide a comprehensive understanding of the statistical relationships between molding parameters and mechanical properties.

### 3.3. Machine Learning (ML)

Traditional performance metrics assessed the predictive capability of mechanical properties like tensile strength, Young’s modulus, and hardness by various machine learning (ML) models for PLA/BNNP composites. The coefficient of determination (R^2^), a measure of the fraction of variance in the target accounted for by the model, and error-oriented measures, such as mean squared error (MSE) and root mean squared error (RMSE), are estimates of the average magnitude of the prediction errors. Furthermore, mean absolute error (MAE) and mean absolute percentage error (MAPE) were applied to quantify error magnitudes in absolute and percentage forms, respectively, by Equations (17)–(22) [62,63]. Both of these metrics provide an overall assessment framework for estimating the accuracy and reliability of every ML model for predicting material hardness based on input process parameters.

(19) $$R^{2}=1-\frac{\sum_{i=1}^{n}{{(O}_{i}-{\bar{O}}_{i})}^{2}}{\sum_{i=1}^{n}{{(O}_{i}-\bar{O})}^{2}}$$

(20) $$MSE=\frac{1}{n}\sum_{i=1}^{n}{{(O}_{i}-{\bar{O}}_{i})}^{2}$$

(21) $$RMSE=\sqrt{MSE}=\sqrt{{{(O}_{i}-{\bar{O}}_{i})}^{2}}$$

(22) $$MAE=\frac{1}{n}\sum_{i=1}^{n}\left|O_{i}-{\bar{O}}_{i}\right|$$

(23) $$MAPE=\frac{1}{n}\sum_{i=1}^{n}\left|\frac{O_{i}-{\bar{O}}_{i}}{O_{i}}\right|\times 100$$

Here, n is the number of trials, and $O_{i}$ and $\bar{O}$ are the actual values and the mean of the actual values, respectively; similarly, ${\bar{O}}_{i}$ is the predicted value.

#### 3.3.1. Linear Regression (LR)

**Figure 16:**
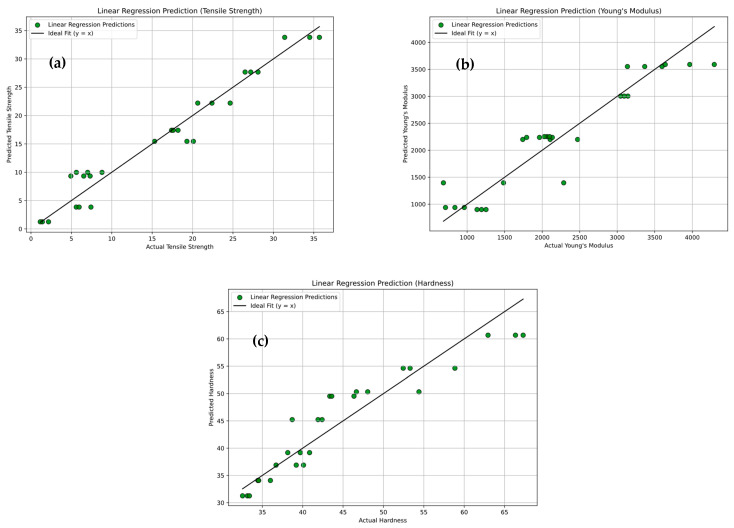
Experimental vs. Linear regression prediction (sector plots). (**a**) Tensile strength, (**b**) Young’s modulus, and (**c**) hardness.

**Figure 17:**
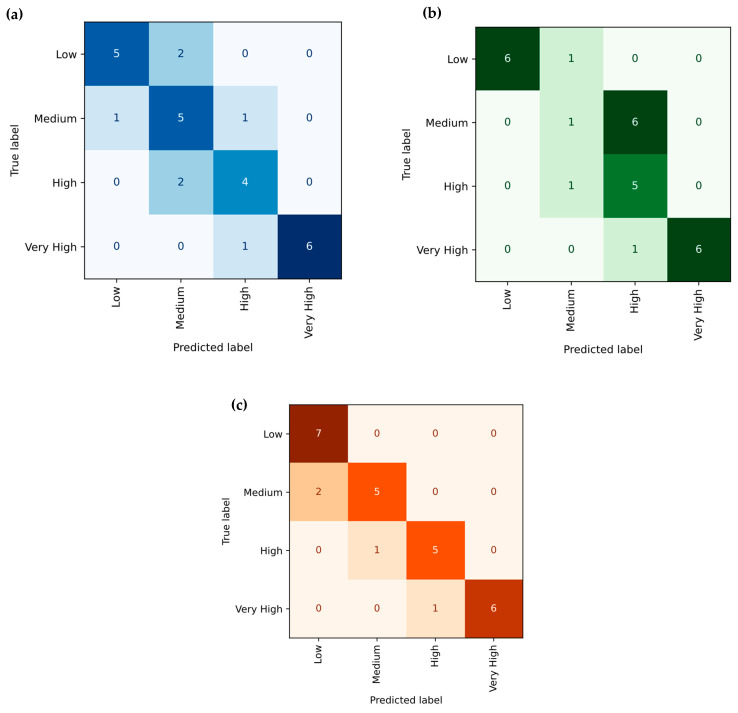
Confusion matrix of actual vs. linear regression predicted; (**a**) tensile strength, (**b**) Young’s modulus, and (**c**) hardness.

Figure 16a–c presents the performance of the linear regression model in predicting the tensile strength, Young’s modulus, and hardness of PLA/BNNP composites, where predicted values on the Y-axis are compared with experimentally measured values on the X-axis. Green dots indicate individual observations, while the black diagonal line (y = x) represents an ideal fit, where predictions perfectly match actual measurements. The model explains 95.31%, 88.66%, and 88.17% of the variance in tensile strength, Young’s modulus, and hardness, respectively. For tensile strength, it achieved an MSE of 5.1378, an RMSE of 2.2667, an MAE of 1.7496, and an MAPE of 20.7553%; for Young’s modulus, an MSE of 118,384.0185, an RMSE of 344.0698, an MAE of 265.8025, and an MAPE of 16.1981%; and for hardness, an MSE of 11.834, an RMSE of 3.44, an MAE of 2.8629, and an MAPE of 6.239%. These results demonstrate that the linear regression model offers high predictive accuracy, with strong agreement between predicted and actual values for both tensile strength and Young’s modulus, and for hardness, moderate prediction of mechanical properties. Figure 17a–c presents confusion matrices for the linear regression model, predicting tensile strength, Young’s modulus, and hardness of PLA/BNNP composites, classified into four levels, including Low, Medium, High, and Very High based on quartile thresholds of the respective datasets. For tensile strength in Figure 17a, the diagonal entries (5, 5, 4, 6) indicate 20 correct predictions out of 27, with errors including two Low predicted as Medium, one Medium as Low, one Medium as High, and one High as Medium. For Young’s modulus in Figure 17b, the diagonal entries (6, 1, 5, 6) correspond to 18 correct predictions out of 27, with errors comprising one Low predicted as Medium, three Medium as High, one Medium as Very High, one High as Medium, and one High as Very High. For hardness in Figure 17c, the diagonal entries (7, 5, 5, 6) yield 23 correct predictions out of 27, with one Low predicted as Medium, one Medium as High, and one High as Very High. Misclassifications were confined to adjacent categories, reflecting the model’s tendency toward near-correct predictions, with comparatively better performance in identifying Low and Very High levels.

#### 3.3.2. Support Vector Regression (SVR)

**Figure 18:**
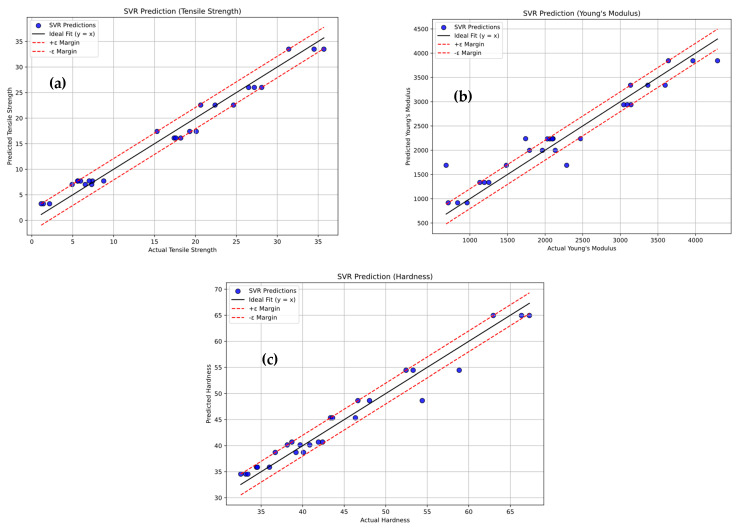
Experimental vs. Support Vector Regression prediction (sector plots). (**a**) Tensile strength, (**b**) Young’s modulus, and (**c**) hardness.

**Figure 19:**
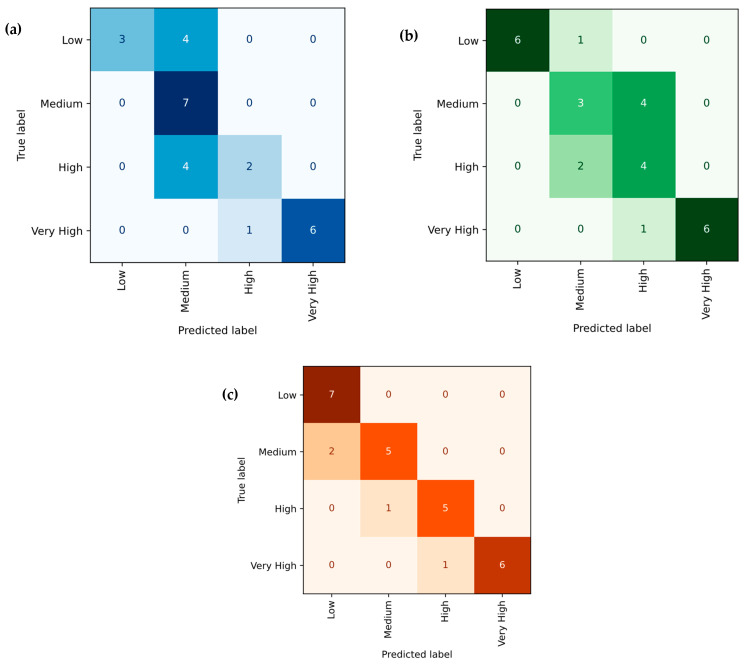
Confusion matrix of actual vs. Support Vector Regression predicted; (**a**) tensile strength, (**b**) Young’s modulus, and (**c**) hardness.

Figure 18a–c illustrates the Support Vector Regression (SVR) model’s prediction performance for tensile strength, Young’s modulus, and hardness of PLA/BNNP composites, with actual values (true labels) on the x-axis and predicted values on the y-axis. Each blue dot represents a data point, while the solid black line (y = x) denotes an ideal prediction. The dashed lines mark the ±ε margin, representing the model’s tolerance range. Notably, most data points lie exactly on or very close to these margins, indicating that the SVR model achieved a highly regularized fit, adhering closely to permissible error limits. For tensile strength, the model achieved an R^2^ of 97.46%, MSE of 2.782, RMSE of 1.668, MAE of 1.507, and MAPE of 24.39%. For Young’s modulus, the results were R^2^ of 91.33%, MSE of 90,478.5, RMSE of 300.796, MAE of 223.947, and MAPE of 14.959%. For hardness, the values were R^2^ of 95.857%, MSE of 4.145, RMSE of 2.036, MAE of 1.695, and MAPE of 3.76%. These results demonstrate the SVR model’s strong predictive accuracy and reliability for mechanical property estimation. Figure 19a–c shows the predictive accuracy of tensile strength, Young’s modulus, and hardness of the Support Vector Regression (SVR) model, respectively, for four classes, e.g., Low, Medium, High, and Very High. Figure 19a presents the tensile strength obtained by 18 experiments out of 27. The diagonal entries (3, 7, 2, 6) indicate 18 correct predictions out of 27, with errors including four Low predicted as Medium, four High as Medium, and one very High as High. For Young’s modulus in Figure 19b, the diagonal entries (6, 3, 4, 6) correspond to 19 correct predictions out of 27, with errors comprising one Low predicted as Medium, three Medium as High, one Medium as Very High, one High as Medium, and one High as Very High. For hardness in Figure 19c, the diagonal entries (7, 5, 5, 6) yield 23 correct predictions out of 27, with one Low predicted as Medium, four Medium as High, two High as Medium, and one very High as High. Misclassifications were confined to adjacent categories, reflecting the model’s tendency toward near-correct predictions, with comparatively better performance in identifying Low and Very High levels.

#### 3.3.3. Random Forest Regression (RFR)

**Figure 20:**
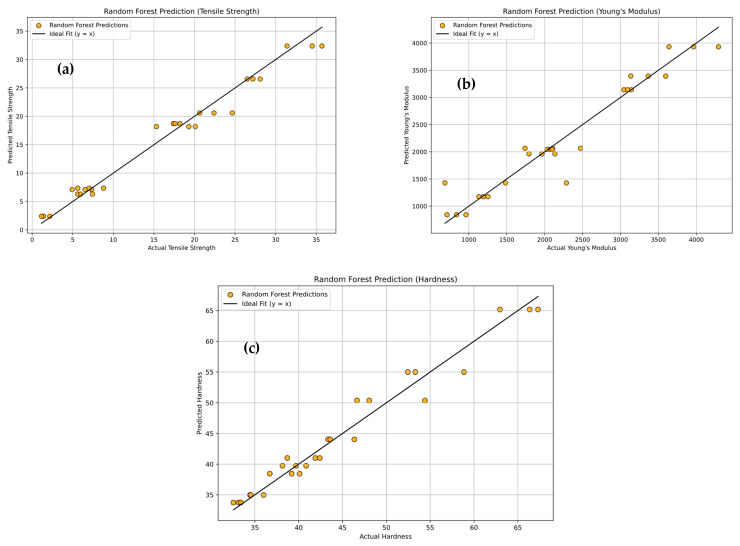
Experimental vs. Random Forest Regression prediction (sector plots). (**a**) Tensile strength, (**b**) Young’s modulus, and (**c**) hardness.

**Figure 21:**
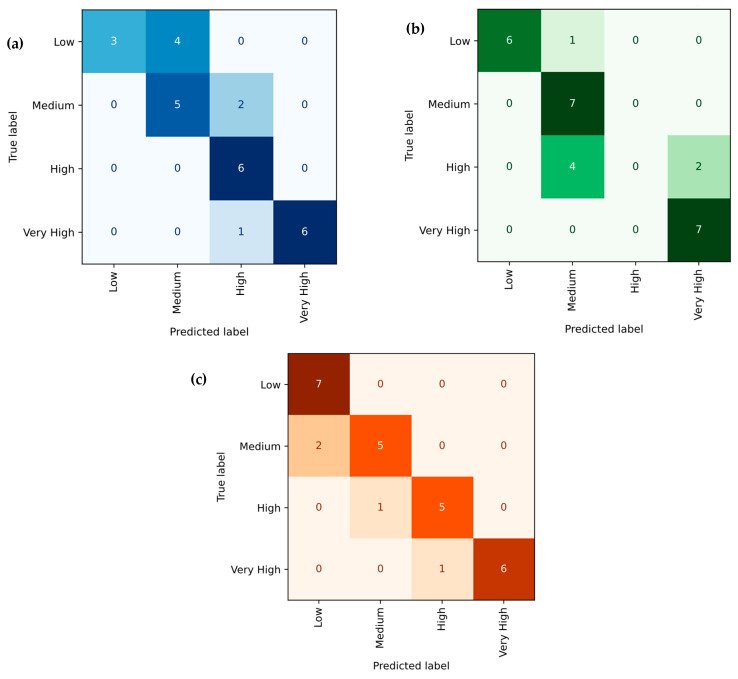
Confusion matrix of actual vs. Random Forest Regression predicted; (**a**) tensile strength, (**b**) Young’s modulus, and (**c**) hardness.

Figure 20a–c presents the predictive performance of the Random Forest Regression (RFR) model for tensile strength, Young’s modulus, and hardness of PLA/BNNP composites. The scatter plots display experimental values (x-axis) against model-predicted values (y-axis), with orange markers representing individual data points. The solid black line (y = x) denotes the ideal prediction, while the proximity of the majority of points to this line and indicates a strong fit [64]. Minor deviations observed in certain regions suggest localized prediction errors, though the overall trend is well-captured. Quantitatively, the tensile strength model achieved R^2^ = 97.63%, MSE = 2.598, RMSE = 1.611, MAE = 1.279, and MAPE = 16.034%. For Young’s modulus, the model yielded R^2^ = 92.88%, MSE = 74,278.04, RMSE = 272.54, MAE = 169.559, and MAPE = 10.685%. Hardness predictions resulted in R^2^ = 96.367%, MSE = 3.634, RMSE = 1.906, MAE = 1.5835, and MAPE = 3.414%. The high R^2^ values and relatively low error metrics across all three properties confirm the RFR model’s robust predictive capacity, with only minor dispersion around the ideal fit. Figure 21a–c presents the confusion matrices for the Random Forest Regression (RFR) model’s classification of tensile strength, Young’s modulus, and hardness of PLA/BNNP composites into four levels: Low, Medium, High, and Very High. In Figure 21a, for tensile strength, the diagonal entries (3, 5, 6, 6) indicate 20/27 correct predictions, with misclassifications confined to adjacent categories [65]. In Figure 21b, for Young’s modulus, it shows diagonal values (6, 7, 0, 7) also totalling 20/27 correct, though high-level predictions were often shifted to neighboring categories. In Figure 21c, for hardness, diagonal values (7, 5, 5, 6) again yield 23/27 correct predictions, with all errors limited to nearby levels. Overall, the RFR model demonstrates moderate classification accuracy, with particularly strong performance in identifying Low and Very High property levels, and misclassifications generally restricted to adjacent classes, indicating near-accurate predictions.

#### 3.3.4. Gradient Boosting Regression (GBR)

**Figure 22:**
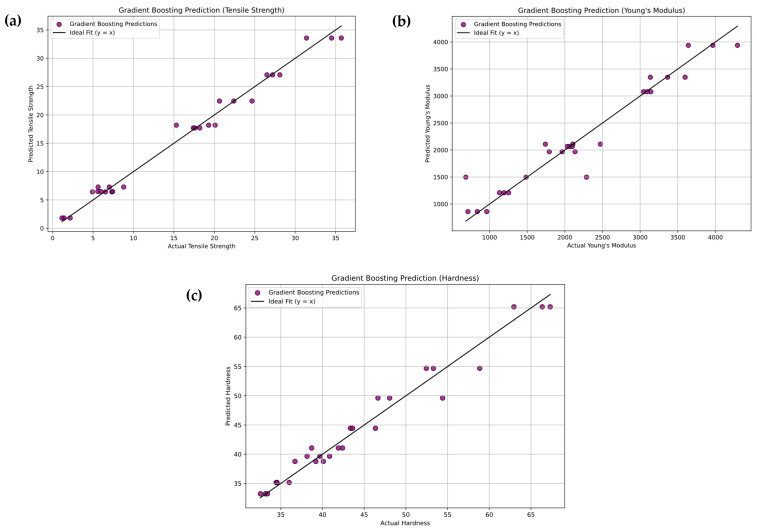
Experimental vs. Gradient Boosting Regression prediction (sector plots). (**a**) Tensile strength, (**b**) Young’s modulus, and (**c**) hardness.

**Figure 23:**
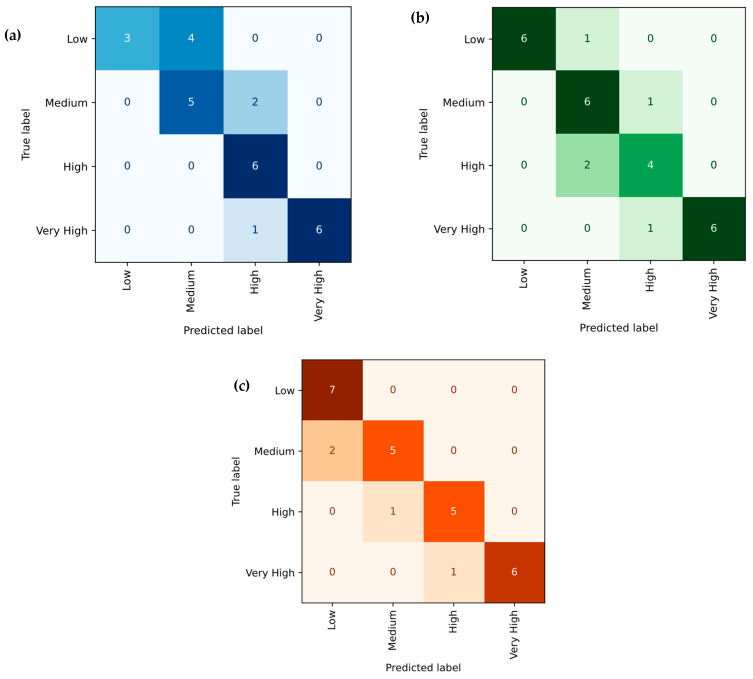
Confusion matrix of actual vs. Gradient Boosting Regression predicted; (**a**) tensile strength, (**b**) Young’s modulus, and (**c**) hardness.

Figure 22a–c illustrates the Gradient Boosting Regression (GBR) model’s predictive performance for tensile strength, Young’s modulus, and hardness of PLA/BNNP composites. Experimental values (x-axis) are plotted against predicted values (y-axis), with purple markers representing data points. The solid black diagonal line (y = x) indicates perfect prediction, and the close clustering of most points around this line reflects strong model accuracy, with only minor deviations observed. For tensile strength, the model achieved R^2^ = 98.51%, MSE = 1.631, RMSE = 1.277, MAE = 1.019, and MAPE = 11.605%. For Young’s modulus, results were R^2^ = 92.97%, MSE = 73,430.29, RMSE = 270.98, MAE = 164.791, and MAPE = 10.827%. For hardness, the values were R^2^ = 96.49%, MSE = 3.5117, RMSE = 1.874, MAE = 1.506, and MAPE = 3.210%. These results confirm the GBR model’s high predictive capability, with slightly stronger performance in modulus prediction. Figure 23a–c shows the Gradient Boosting model’s confusion matrices for tensile strength, Young’s modulus, and hardness of PLA/BNNP composites, classified into Low, Medium, High, and Very High levels. For tensile strength, diagonal values (3, 5, 6, 6) yielded 20/27 correct predictions, with two adjacent-category misclassifications. Young’s modulus achieved diagonal values (6, 6, 4, 6), with 22/27 correct and errors limited to neighboring classes. Hardness results (7, 5, 5, 6) gave 23/27 correct, with a single Low misclassified as Medium. Across all properties, misclassifications were confined to adjacent categories, indicating strong classification accuracy and the model’s ability to closely approximate true class boundaries.

#### 3.3.5. Extreme Gradient Boosting (XG-Boosting)

**Figure 24:**
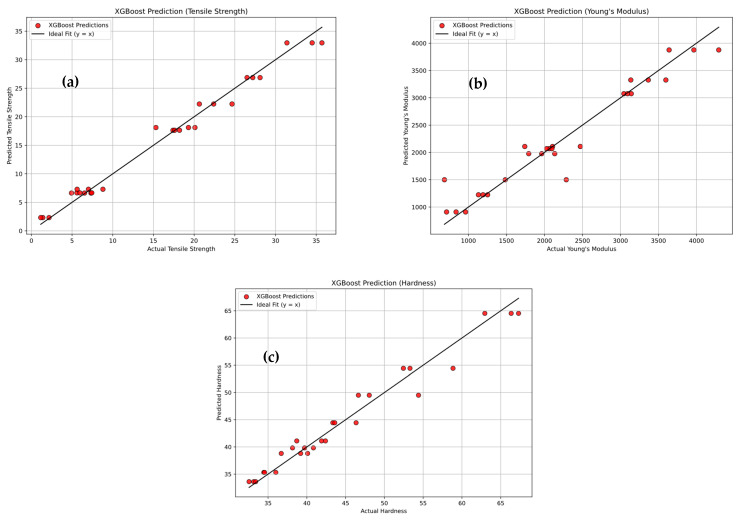
Experimental vs. XG-Boost Regression prediction (sector plots). (**a**) Tensile strength, (**b**) Young’s modulus, and (**c**) hardness.

**Figure 25:**
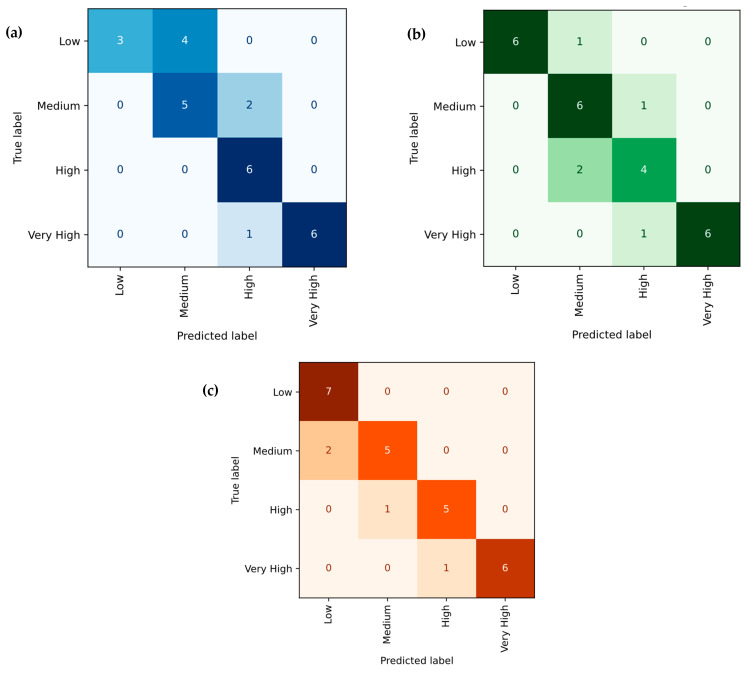
Confusion matrix of actual vs. XG-Boost Regression predicted; (**a**) tensile strength, (**b**) Young’s modulus, and (**c**) hardness.

Figure 24a–c depicts the predictive performance of the XG-Boost Regression model for tensile strength, Young’s modulus, and hardness of PLA/BNNP composites. Experimental values (x-axis) are plotted against model predictions (y-axis), with red markers, representing individual data points. The solid black diagonal line (y = x) indicates ideal prediction, and the close clustering of most points around this line reflects high predictive accuracy, with only minor deviations observed. For tensile strength, the model achieved R^2^ = 98.34%, MSE = 1.817, RMSE = 1.348, MAE = 1.092, and MAPE = 14.565%. For Young’s modulus, results were R^2^ = 92.81%, MSE = 75,021.91, RMSE = 273.90, MAE = 170.916, and MAPE = 11.28%. For hardness, values were R^2^ = 96.32%, MSE = 3.682, RMSE = 1.918, MAE = 1.553, and MAPE = 3.325%. These metrics confirm XG-Boost’s robust and highly accurate predictive capability across all three mechanical properties. Figure 25a–c shows the XG-Boost model’s confusion matrices for tensile strength, Young’s modulus, and hardness of PLA/BNNP composites, classified into Low, Medium, High, and Very High levels. For tensile strength, diagonal values (3, 5, 6, 6) yielded 20/27 correct predictions, with two adjacent-category misclassifications [66]. Young’s modulus achieved diagonal values (6, 6, 4, 6), with 22/27 correct and errors limited to neighboring classes. Hardness results (7, 5, 5, 6) gave 23/27 correct, with a single Low misclassified as Medium. Across all properties, misclassifications were confined to adjacent categories, indicating strong classification accuracy and the model’s ability to closely approximate true class boundaries.

**Figure 26:**
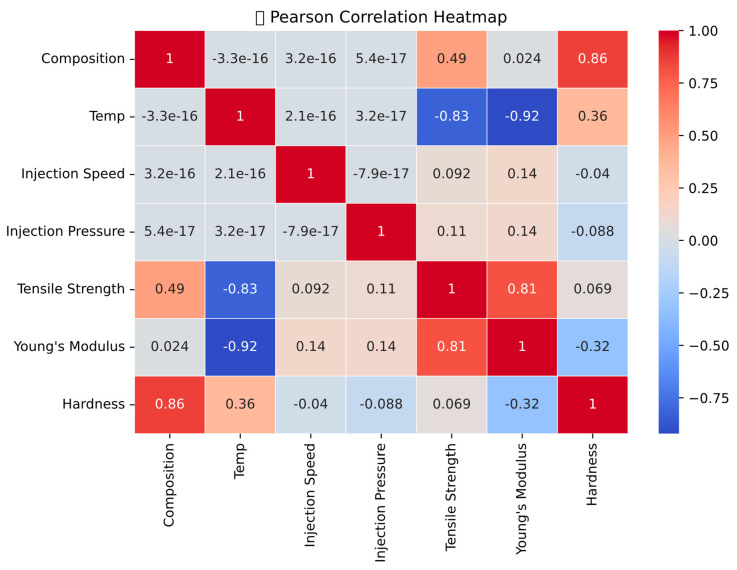
Correlation heatmap.

Figure 26 illustrates the correlation heatmap, showing the relationships between processing parameters and the mechanical properties of PLA/BNNP composites. Tensile strength shows a strong positive correlation (0.81) with Young’s modulus, indicating that these properties change together. Temperature exhibits a high negative correlation with Young’s modulus (−0.92) and tensile strength (−0.83), confirming that increasing temperature reduces both properties. Composition is strongly positively correlated with hardness (0.86) and moderately positively correlated with tensile strength (0.49), suggesting its role in enhancing these properties. Injection speed and injection pressure display weak correlations with the outputs, indicating limited direct influence. Temperature also shows a moderate positive correlation with hardness (0.34), implying a potential improvement in surface properties at the expense of strength and stiffness. Overall, the heatmap highlights temperature as the most influential parameter governing composite performance and provides valuable insight into variable interactions for understanding machine learning model predictions.

**Figure 27:**
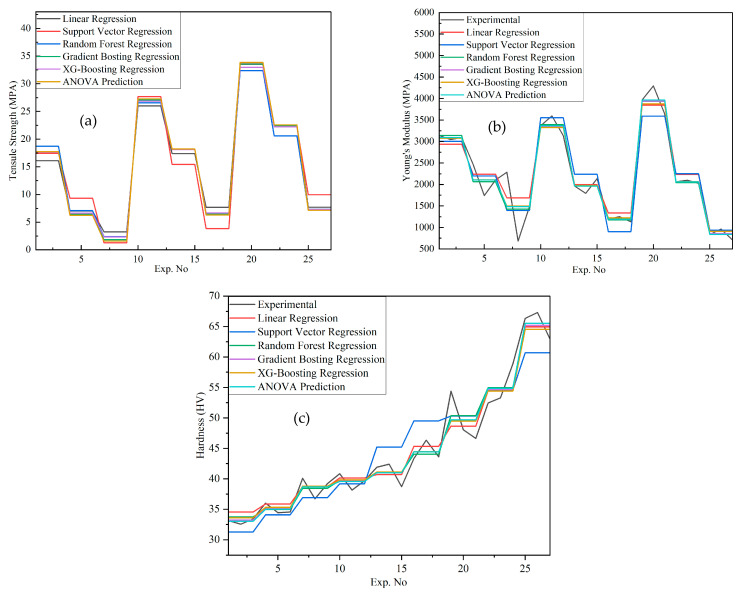
Comparison of experimental results with ANOVA and ML models. (**a**) Tensile strength, (**b**) Young’s modulus, and (**c**) hardness.

Figure 27a–c presents a comparative analysis of tensile strength, Young’s modulus, and hardness predicted by different machine learning models alongside ANOVA and experimental results. Figure 27a shows that the tensile strength predictions obtained from XG-Boosting are in close agreement with the experimental data, with deviations below 10%; for example, in Experiment 1, the predicted value of 17.636 MPa closely matches the experimental tensile strength of 17.733 MPa. Figure 27b illustrates the predictions of Young’s modulus, where XG-Boosting and ANOVA again provide the most accurate estimates, such as a predicted modulus of 3142 MPa for the first experiment, demonstrating strong predictive capability. Figure 27c presents the hardness predictions, in which Random Forest, Gradient Boosting, and XG-Boosting models show minimal deviation from experimental values; for instance, in Experiment No. 27, the predicted hardness of 65.53 HV closely matches the experimental value of 65.33 HV. In contrast, Support Vector Regression exhibits lower prediction confidence across all three figures, likely due to its sensitivity to kernel selection and hyperparameter tuning and its limited ability to capture complex nonlinear interactions. Overall, while all models capture general trends, ensemble-based methods—particularly XG-Boosting—demonstrate superior robustness and accuracy in predicting the mechanical properties of PLA/BNNP composites.

## 4. Conclusions

This study investigates the mechanical performance and predictive modeling of polylactic acid (PLA) reinforced with boron nitride nanoplatelets (BNNPs), fabricated by injection molding. A Taguchi L27 orthogonal array was employed to analyze the effects of composition (PLA, 0.02 wt.%, 0.04 wt.%), injection temperature (135–155 °C), injection speed (50–70 mm/s), and pressure (30–50 bar) on tensile strength, Young’s modulus, and hardness. The experimental outcomes were further analyzed and predicted using ANOVA and advanced machine learning models. The key findings are as follows:

- Reinforcement with 0.04 wt.% BNNP significantly enhanced PLA performance, improving tensile strength by 18.6%, Young’s modulus by 32.7%, and hardness by 20.5% compared to neat PLA.
- Taguchi L27 design revealed that higher BNNP content at optimal injection temperatures enhanced all properties, while excessive temperatures caused a reduction in tensile strength and modulus but an increase in hardness.
- ANOVA showed that processing temperature was the dominant factor for tensile strength (68.88%) and Young’s modulus (86.39%), whereas BNNP content was the major contributor to hardness (78.83%), followed by temperature (13.36%). Injection speed (60 mm/s) and pressure (40 bar) had only minor effects.
- Among the machine learning models used, XGBoost demonstrated the highest predictive accuracy, achieving R^2^ values above 98% for tensile strength, 92–93% for Young’s modulus, and 96% for hardness, with low error metrics (RMSE, MAE, MAPE). These results confirm that XGBoost is the most reliable model for property prediction in this study. However, the other models, such as RFR and GBR, also performed well, albeit with slightly lower accuracy than XGBoost.

The study is constrained by a limited dataset with a narrow range of filler loadings. While the prediction models have shown high accuracy within the range of parameters analyzed, further research with a broader range of filler loadings and different fillers will help to validate and potentially improve the generalization of these machine learning models to a wider range of parameters [67,68].

## Figures and Tables

**Table 1:** Factors and levels for the fabrication process.

S.No	Factors	Units	Level 1	Level 2	Level 3
1	Composition	Wt.%	Pure	0.02	0.04
2	Temperature (temp)	°C	135	145	155
3	Injection Speed	mm/s	50	60	70
4	Injection Pressure	Bar	30	40	50

**Table 2:** Taguchi L27 orthogonal array with experimentally obtained tensile strength and Young’s modulus with S/N ratio.

Exp. No	Composition (Wt.%)	Temperature (°C)	Injection Speed (m/s)	Injection Pressure (bar)	Tensile Strength (MPa)	Young’s Modulus (MPa)	S/N Ratio (Tensile Strength)	S/N Ratio (Young’s Modulus)
1	0	135	50	30	17.4	3142	24.97	69.80
2	0	135	50	30	17.6	3045	24.97	69.80
3	0	135	50	30	18.2	3093	24.97	69.80
4	0	145	60	40	6.53	2472	15.56	66.20
5	0	145	60	40	4.93	1741	15.56	66.20
6	0	145	60	40	7.32	2106	15.56	66.20
7	0	155	70	50	1.41	2287	3.09	60.32
8	0	155	70	50	1.15	684	3.08	60.32
9	0	155	70	50	2.17	1485	3.08	60.32
10	0.02	135	60	50	27.2	3366	28.71	70.50
11	0.02	135	60	50	28.1	3597	28.70	70.50
12	0.02	135	60	50	26.5	3136	28.70	70.50
13	0.02	145	70	30	19.3	1964	25.02	65.79
14	0.02	145	70	30	15.3	1793	25.02	65.79
15	0.02	145	70	30	20.1	2135	25.02	65.79
16	0.02	155	50	40	5.6	1192	15.84	61.50
17	0.02	155	50	40	7.42	1252	15.83	61.50
18	0.02	155	50	40	5.96	1132	15.83	61.50
19	0.04	135	70	40	34.5	3966	30.55	71.90
20	0.04	135	70	40	31.4	4293	30.55	71.90
21	0.04	135	70	40	35.7	3640	30.55	71.90
22	0.04	145	50	50	24.655	2063	27.00	66.28
23	0.04	145	50	50	20.64	2098	27.00	66.28
24	0.04	145	50	50	22.4	2029	27.00	66.28
25	0.04	155	60	30	7.014	837	16.64	58.25
26	0.04	155	60	30	5.62	962	16.64	58.25
27	0.04	155	60	30	8.79	712	16.64	58.25

**Table 3:** Taguchi L27 Orthogonal array with experimentally obtained hardness with S/N ratio.

Exp. No	Composition (Wt.%)	Temperature (°C)	Injection Speed (m/s)	Injection Pressure (bar)	Hardness (HV)	S/N Ratio (Hardness)
1	0	135	50	30	33.15	30.38
2	0	135	50	30	32.55	30.38
3	0	135	50	30	33.4	30.38
4	0	145	60	40	36	30.88
5	0	145	60	40	34.45	30.88
6	0	145	60	40	34.55	30.87
7	0	155	70	50	40.1	31.72
8	0	155	70	50	36.7	31.73
9	0	155	70	50	39.2	31.72
10	0.02	135	60	50	40.85	31.93
11	0.02	135	60	50	38.15	31.94
12	0.02	135	60	50	39.7	31.93
13	0.02	145	70	30	41.9	32.23
14	0.02	145	70	30	42.4	32.23
15	0.02	145	70	30	38.7	32.23
16	0.02	155	50	40	43.35	32.94
17	0.02	155	50	40	46.35	32.94
18	0.02	155	50	40	43.6	32.94
19	0.04	135	70	40	54.4	33.87
20	0.04	135	70	40	48.05	33.87
21	0.04	135	70	40	46.65	33.87
22	0.04	145	50	50	52.45	34.75
23	0.04	145	50	50	53.3	34.75
24	0.04	145	50	50	58.85	34.75
25	0.04	155	60	30	66.35	36.31
26	0.04	155	60	30	67.3	36.31
27	0.04	155	60	30	62.95	36.31

**Table 4:** Tensile strength response table for signal-to-noise ratios and mean.

Level	Signal-to-Noise Ratio				Mean			
	Composition	Temp	Injection Speed	Injection Pressure	Composition	Temp	Injection Speed	Injection Pressure
1	14.54	28.08	22.60	22.21	8.52	26.28	15.54	14.36
2	23.19	22.53	20.31	20.65	17.27	15.68	13.55	15.48
3	24.73	11.86	19.56	19.60	21.19	5.01	17.89	17.13
Delta	10.19	16.22	3.05	2.62	12.66	21.27	4.33	2.76
Rank	2	1	3	4	2	1	3	4

**Table 5:** Young’s modulus response table for signal-to-noise ratios and mean.

Level	Signal-to-Noise Ratio				Mean			
	Composition	Temp	Injection Speed	Injection Pressure	Composition	Temp	Injection Speed	Injection Pressure
1	65.45	70.74	65.87	64.62	2228	3475	2116	1965
2	65.93	66.10	64.99	66.54	2174	2045	2103	2422
3	65.49	60.03	66.01	65.71	2289	1171	2472	2305
Delta	0.49	10.71	1.02	1.92	115	2304	369	457
Rank	4	1	3	2	4	1	3	2

**Table 6:** Hardness response table for signal-to-noise ratios and mean.

Level	Signal-to-Noise Ratio				Mean			
	Composition	Temp	Injection Speed	Injection Pressure	Composition	Temp	Injection Speed	Injection Pressure
1	30.99	32.06	32.69	32.98	35.57	40.77	44.11	46.52
2	32.37	32.62	33.04	32.56	41.67	43.62	46.70	43.04
3	34.98	33.66	32.61	32.81	56.70	49.54	43.12	44.37
Delta	3.99	1.60	0.43	0.41	21.13	8.78	3.58	3.48
Rank	1	2	3	4	1	2	3	4

**Table 7:** Analysis to optimize processing parameters to obtain tensile strength using ANOVA.

Source	DF	Seq SS	Adj SS	Adj MS	F-Value	*p*-Value	Contribution
Composition	2	757.25	757.25	378.62	157.60	0.004 × 10^−9^	25.61%
Temp	2	2036.69	2036.69	1018.35	423.89	0.007 × 10^−13^	68.88%
Injection Speed	2	84.80	84.80	42.40	17.65	0.006 × 10^−2^	2.87%
Injection Pressure	2	34.87	34.87	17.44	7.26	0.005	1.18%
Error	18	43.24	43.24	2.40			1.46%
Total	26	2956.86					100.00%
R-sq = 98.54%, R-sq.(adj) = 97.89%							

**Table 8:** Analysis to optimize processing parameters to obtain Young’s modulus using ANOVA.

Source	DF	Seq SS	Adj SS	Adj MS	F-Value	*p*-Value	Contribution
Composition	2	59,343.00	59,343	29,671	0.27	0.766	0.21%
Temp	2	24,352,056	24,352,056	12,176,028	110.95	0.007 × 10^−8^	86.39%
Injection Speed	2	787,749	787,749	393,874	3.59	0.048	2.79%
Injection Pressure	2	1,013,947	1,013,947	506,973	4.62	0.024	3.60%
Error	18	1,975,468	1,975,468	109,748			7.01%
Total	26	28,188,563					100.00%
R-sq = 92.99%, R-sq.(adj) = 89.88%							

**Table 9:** Analysis to optimize processing parameters to obtain hardness using ANOVA.

Source	DF	Seq SS	Adj SS	Adj MS	F-Value	*p*-Value	Contribution
Composition	2	2129.49	2129.49	1064.74	203.66	0.004 × 10^−10^	78.83%
Temp	2	360.83	360.83	180.41	34.51	0.006 × 10^−4^	13.36%
Injection Speed	2	61.44	61.44	30.72	5.88	0.011	2.27%
Injection Pressure	2	55.47	55.47	27.73	5.3	0.015	2.05%
Error	18	94.1	94.1	5.23			3.48%
Total	26	2701.33					100.00%
R-sq = 96.52%, R-sq.(adj) = 94.97%							

## Data Availability

The original contributions presented in this study are included in the article/Supplementary Materials. Further inquiries can be directed to the corresponding author.

## Supplementary Materials

The following supporting information can be downloaded at https://www.mdpi.com/article/10.3390/polym18020185/s1; Figure S1. Tensile testing setup using the H10KL Universal Testing Machine with Testing sample specifications; Figure S2. Hardness testing setup using the Vickers hardness tester; Figure S3. Feature Importance in Injection Molding Parameters Based on ANOVA: p-Values and F-Values for Mechanical Properties.
