# Supplementary material for: Data-Driven AI Approach for Optimizing Processes and Predicting Mechanical Properties of Boron Nitride Nanoplatelet-Reinforced PLA Nanocomposites
Source: Polymers (Basel). 2026 Jan 9;18(2):185. doi: 10.3390/polym18020185 (PMC12845918; doi:10.3390/polym18020185)
Supplement: Supplementary file 1 [file polymers-18-00185-s001.zip › polymers-4017726-supplementary.pdf]

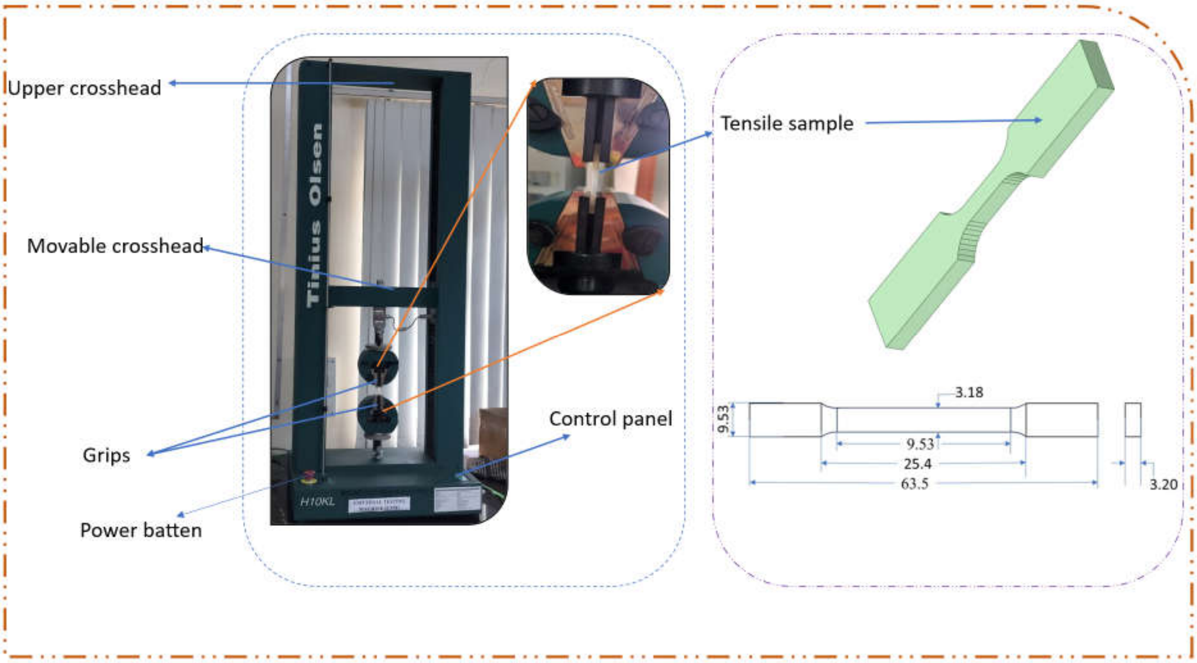

Figure S1. Tensile testing setup using the H10KL Universal Testing Machine with Testing sample specifications.

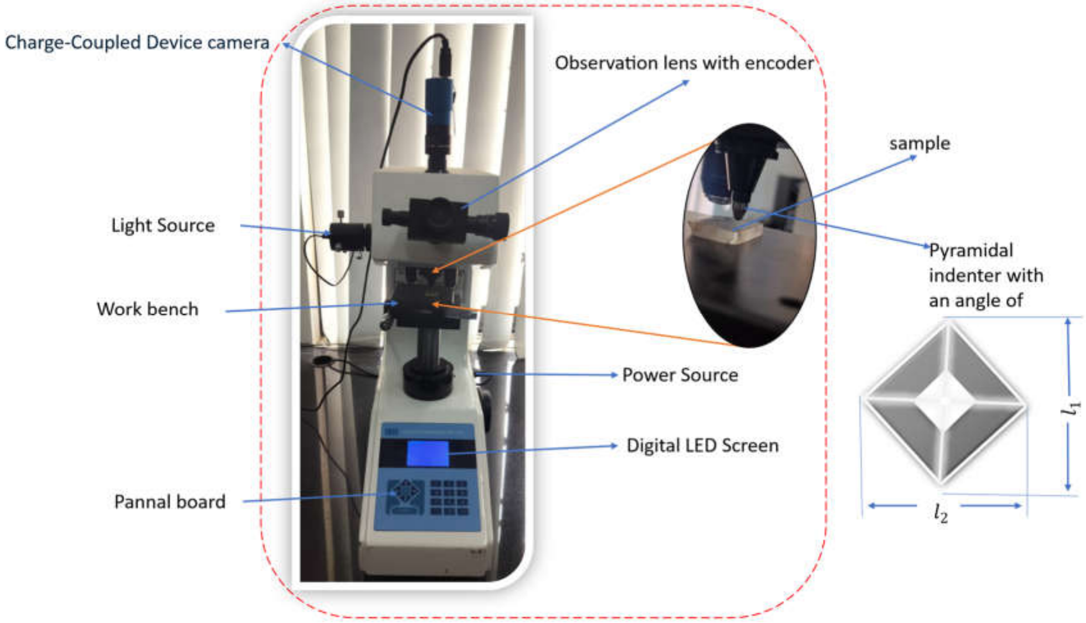

Figure S2. Hardness testing setup using the Vickers hardness tester.

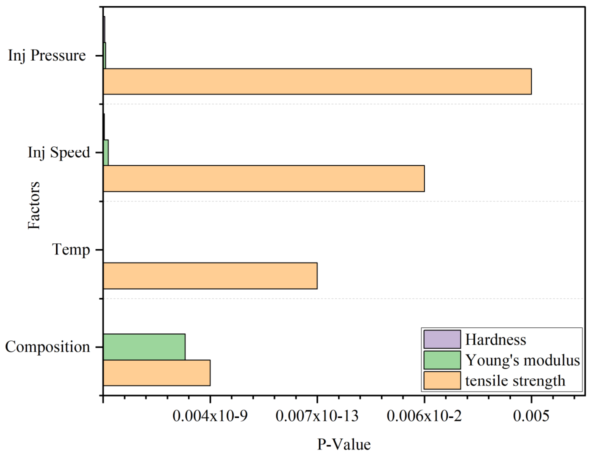

(**a**)

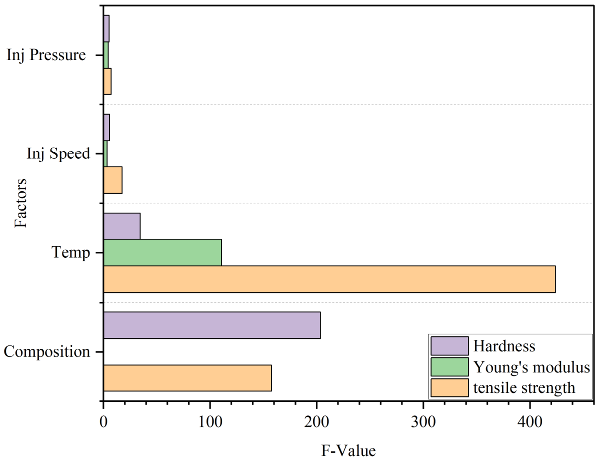

(**b**)

Figure S3. Feature Importance in Injection Molding Parameters Based on ANOVA (a) p-Values and (b) F-Values for Mechanical Properties
